# HDAC1 dysregulation promotes pro-inflammatory microglial activation and aggravates post-stroke neuroinflammation

**DOI:** 10.1080/07853890.2025.2597624

**Published:** 2025-12-12

**Authors:** Jui-Shen Chen, Hao-Kuang Wang, Yu-Ting Su, Yu-Cheng Ho, Cheng-Loong Liang, Yung-Kuo Lee, Tian-Huei Chu, Yun-Shin Lin, Cheng-Chun Wu

**Affiliations:** ^a^ Department of Neurosurgery, E-DA Hospital, I-Shou University, Kaohsiung City, Taiwan; ^b^ Graduate Institute of Medicine, College of Medicine, I-Shou University, Kaohsiung City, Taiwan; ^c^ Department of Obstetrics and Gynecology, Kaohsiung Chang Gung Memorial Hospital and Chang Gung University College of Medicine, Kaohsiung City, Taiwan; ^d^ School of Medicine, College of Medicine, I-Shou University, Kaohsiung City, Taiwan; ^e^ Medical Laboratory, Medical Education and Research Center, Kaohsiung Armed Forces General Hospital, Kaohsiung City, Taiwan; ^f^ Division of Experimental Surgery Center, Department of Surgery, Tri-Service General Hospital, National Defense Medical University, Taipei, Taiwan; ^g^ School of Medicine, National Defense Medical University, Taipei, Taiwan; ^h^ Institute of Medical Science and Technology, National Sun Yat-sen University, Kaohsiung, Taiwan; ^i^ Department of Psychiatry, Kaohsiung Armed Forces General Hospital, Kaohsiung city, Taiwan

**Keywords:** HDAC1, stroke, microglia, inflammation

## Abstract

**Background:**

Histone deacetylase 1 (HDAC1) is a key epigenetic regulator involved in DNA repair and neuronal survival. While HDAC1 downregulation has been implicated in ischemic brain injury, its role in regulating microglial functional shift and neuroinflammation remains unclear. This study aimed to investigate how HDAC1 dysfunction influences microglial activation states and contributes to neuroinflammatory processes in ischemic stroke.

**Methods:**

Using a rat model of endothelin-1–induced focal cerebral ischemia, HDAC1 knockdown was achieved *via* stereotactic co-injection of HDAC1 siRNA. Immunofluorescence, Western blotting, ELISA, and oxidative stress assays were performed to assess neuroinflammation, microglial polarization, and related signalling pathways. In parallel, HDAC1 was silenced in HMC3 human microglial cells, with or without IFN-γ stimulation, to evaluate transcriptional responses associated with pro-inflammatory activation. Finally, HDAC1 was selectively reactivated *in vivo* and *in vitro* using Compound 5104434 to assess behavioural, neuroinflammatory and mechanistic effects after ischaemic or inflammatory injury.

**Results:**

HDAC1 knockdown *in vivo* led to a pronounced shift towards a pro-inflammatory microglial activation, evidenced by increased CD86 expression, along with elevated levels of IL-1β, IL-6, TNF-α, ROS, LDH and MMP activity. T-cell infiltration was also significantly enhanced. *In vitro*, HDAC1 deficiency sensitized microglia to IFN-γ, further amplifying the expression of pro-inflammatory genes. Mechanistically, HDAC1 knockdown activated the NF-κB pathway and its downstream effectors *MAP3K8, AP-1* and *SAT1,* while IFN-γ stimulation predominantly drove STAT3 phosphorylation. Notably, pNF-κB was upregulated even in the absence of exogenous stimulation, indicating that HDAC1 intrinsically suppresses pro-inflammatory signalling. HDAC1 enzymatic reactivation by compound 5104434 promotes functional recovery and suppresses NF-κB–driven microglial activation after stroke.

**Conclusion:**

HDAC1 acts as a key repressor of NF-κB–driven pro-inflammatory microglial activation and neuroinflammation in stroke. Its loss exacerbates inflammatory cascades, immune cell infiltration, and neuronal injury, underscoring HDAC1 as a potential therapeutic target for limiting secondary brain damage after ischaemic stroke.

## Background

Histone deacetylase 1 (HDAC1) is a key epigenetic regulator involved in chromatin remodelling, DNA damage repair and neuronal survival [1,2]. In the central nervous system (CNS), HDAC1 plays a crucial role in maintaining genomic stability by modulating DNA repair mechanisms [3–5]. Its dysregulation has been implicated in various neurodegenerative disorders, where defective DNA repair contributes to disease progression. For example, in Alzheimer’s disease (AD), HDAC1 deficiency impairs the repair of oxidative DNA lesions, leading to neuronal DNA damage and cognitive decline [6,7]. Similarly, in amyotrophic lateral sclerosis (ALS), disrupted DNA repair mechanisms have been linked to motor neuron degeneration [8].

Our previous studies demonstrated that HDAC1 expression and enzymatic activity are significantly reduced following ischemic stroke in a rat model of brain ischemia [9]. HDAC1 inhibition exacerbated stroke outcomes, increasing infarct volume, neuronal loss, and reactive oxygen species (ROS) production. Moreover, it impaired motor and functional recovery [9], highlighting its neuroprotective role in ischemic brain injury. In parallel, we identified pharmacological approaches to restore HDAC1 activity, which conferred neuroprotection against oxygen-glucose deprivation (OGD)-induced neuronal injury. HDAC1 reactivation preserved neurite morphology and neuronal viability *in vitro* and significantly reduced infarct volume while improving neurological function *in vivo* [10].

In the brain, microglia, the resident immune cells of the CNS, exhibit a spectrum of activation states, ranging from the pro-inflammatory phenotype, which exacerbates neuronal damage, to the anti-inflammatory phenotype, which promotes tissue repair [11,12]. Our recent findings suggest that HDAC1 dysfunction drives microglial reactivation, aggravating neuroinflammation and blood-brain barrier (BBB) disruption in stroke [13]. Specifically, HDAC1 dysfunction disrupts neuronal homeostasis, leading to excessive DNA damage accumulation, neuronal apoptosis and exacerbation of neuroinflammation and BBB damage [9,13].

Despite these insights, the precise molecular mechanisms by which HDAC1 regulates microglial activation remain unclear. Understanding how HDAC1 dysfunction promotes neuroinflammation through pro-inflammatory microglial activation is critical for developing targeted therapeutic strategies for ischemic stroke. In this study, we investigate the role of HDAC1 in microglial phenotypic transitions and its impact on stroke-induced neuroinflammation. We hypothesize that impaired HDAC1 function exacerbates post-stroke inflammation by shifting microglia towards a pro-inflammatory state, thereby worsening neurological outcomes. Using both *in vitro* and *in vivo* models, we assess the effects of HDAC1 inhibition on microglial activation, inflammatory signalling pathways and functional recovery.

## Materials and methods

### Animal experiments and drug administration

All animal experiments were conducted in accordance with the ARRIVE guidelines 2.0 for reporting animal research (https://arriveguidelines.org), and approved by the Institutional Animal Care and Use Committee (IACUC) of I-Shou University and E-Da Hospital (IACUC-ISU-109031, 2021/08/01 to 2025/07/31; IACUC-EDAH-111031, 2023/01/01 to 2024/01/01). Male Sprague-Dawley (SD) rats were purchased from Lasco Biotechnology (Taipei, Taiwan), and a total of 64 rats were included in this study. Animals were specific-pathogen-free (SPF) and housed under standard conditions (12-hour light/dark cycle, ad libitum access to food and water). All animals were non-transgenic and immunocompetent, with no history of prior surgical, pharmacological, or behavioural interventions before the study. Upon arrival, animals were allowed to acclimate for at least 7 days before experimental procedures commenced.

For Sample-size justification, our primary endpoint was the modified Neurological Severity Score (mNSS). Based on pilot data, we assumed a between-animal variability of SD ≈ 1.7 points and a minimally important difference (MID) of 30%. An a priori power analysis for a one-way ANOVA (k = [3/4/5] groups, α = 0.05, 1–β = 0.80, equal allocation) supported a sample size of *n* = 8 per group. For planned comparisons versus control (Dunnett), this sample size retains ≥80% power to detect differences ≥ the MID corresponding to approximately Cohen’s *d* ≈ 1.5. Calculations were performed in G*Power v3.1. With *n* = 8 per group and α = 0.05, a *k* = 4 one-way ANOVA has ∼80% power to detect an omnibus effect of about Cohen’s *f* ≈ 0.63 (≈ η^2^ ≈ 0.28). Animals were randomly assigned to one of four groups: Sham, Sham + HDAC1 knockdown (KD), Stroke, and Stroke + HDAC1 KD **(***n* = 8 per group). This sample size was chosen to ensure sufficient statistical power for detecting biologically meaningful differences, while also providing adequate tissue for both brain sectioning-based histological analysis and biochemical assays such as protein extraction and Western blotting.

To establish an ischemia/reperfusion injury model, a stereotactic intracerebral injection of endothelin-1, a highly effective vasoconstrictor, was performed [9,10]. The male rats, aged eight weeks and weighing 250–300 g, were randomly assigned to one of three groups: sham control (HBSS injection), endothelin-1 cerebral microinjection, or endothelin-1 combined with HDAC1 siRNA microinjection. For the induction of cerebral ischemia/reperfusion injury, surgery was performed under isoflurane anaesthesia (4–5% for induction, 1.5–2.5% for maintenance) delivered *via* a nose cone. Buprenorphine (0.05 mg/kg, s.c.) was administered preoperatively and every 8–12 h for 48 h post-surgery for pain control, in accordance with American Veterinary Medical Association (AVMA) Guidelines (2020). For stereotactic injection, 2 µL of 100 pM endothelin-1 (Sigma, E7764; St. Louis, MO, USA) was stereotactically injected at specific coordinates based on the brain atlas: AP 0, ML +2.5, DV −2.3; AP +2.3, ML +2.5, DV −2.3; AP +0.7, ML +3.8, DV −7.0. HDAC1 siRNA was pre-mixed with endothelin-1 before injection, resulting in 1 μl of HDAC1 siRNA and 2 μl of endothelin-1 within a total 3 μL volume. All injections were performed in synchrony to ensure uniform drug administration. Seventy-two hours post-surgery, rats were euthanized, and brain tissues were collected for Western blot analysis and immunostaining. At study endpoints, rats were euthanized by CO_2_ inhalation with gradual fill (30–70% chamber volume/min) per AVMA Guidelines (2020), followed by a secondary physical method (e.g. thoracotomy) to ensure death.

Animals were included in the study if they exhibited stable baseline physiological status and recovered adequately from surgical procedures. Exclusion criteria were defined a priori and included: (1) death during or shortly after surgery, (2) severe intraoperative complications, and (3) failure of HDAC1 knockdown as confirmed by post hoc molecular validation. Animals with significant tissue damage that precluded accurate sectioning or protein extraction were also excluded. No animals were excluded based on the outcome of the experimental measurements. For exclusions during analysis, all animals that survived and met the predefined criteria were included in the final analysis. For all four groups, no animals or data points were excluded from analysis. Data from each animal were independently analysed and reported.

To minimize potential confounding factors, animals were randomly assigned to experimental groups and surgical procedures were performed in a randomized order. All treatments and measurements were conducted by investigators blinded to group assignments. Cage location within the animal facility was rotated and balanced to avoid environmental bias related to housing conditions (e.g. light exposure, noise, airflow). Additionally, behavioural and biochemical assessments were performed at similar times of day to reduce circadian variability. No systematic bias was introduced based on treatment order or cage location.

### HDAC1 siRNA knockdown in vivo and in vitro

HDAC1 knockdown in rat brain was achieved using *in vivo*–ready siRNAs (Ambion, Thermo Fisher Scientific; Catalogue #4457308, Assay ID s150967) delivered by stereotaxic injection together with endothelin-1 to induce focal ischemia. Several candidate siRNA sequences against HDAC1 were tested, and the sequence with the most effective knockdown was used for subsequent experiments (Supplementary Figure 1). A negative control siRNA (Catalogue #4457287, Thermo Fisher Scientific) was used as control. Knockdown efficiency was confirmed by Western blot and immunofluorescent staining, showing reduced HDAC1 protein expression relative to negative control, while HDAC2 and HDAC3 levels remained unchanged (Supplementary Figures 2–4). For *in vitro* HDAC1 KD, the human microglial cell line HMC3 was transfected with HDAC1 siRNA (Thermo Fisher Scientific; Catalogue #4392420, Assay IDs S74) using Lipofectamine RNAiMAX (Thermo Fisher Scientific) according to the manufacturer’s protocol. Knockdown efficiency was assessed by Western blot, confirming reduced HDAC1 expression compared to untreated and negative controls (Supplementary Figure 5). The target sequences, target exons, and siRNA locations are available on the manufacturer’s official product webpage, referenced by the catalogue numbers and assay IDs provided in this study.

**Figure 2.**
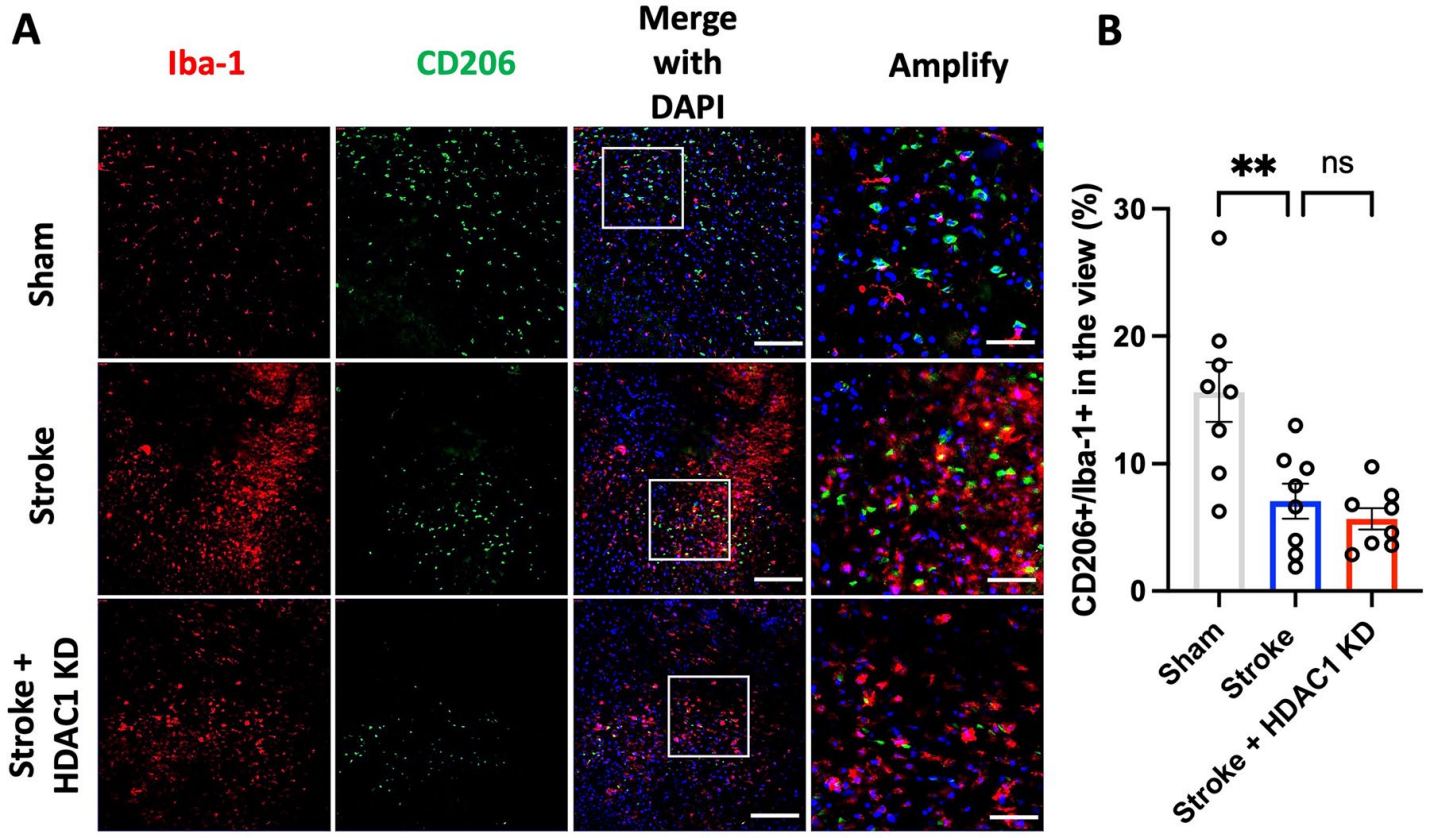
Ischaemia insult suppresses anti-inflammatory microglial activation after stroke. (A) Representative immunofluorescence images showing Iba-1 (red), CD206 (green, M2 marker), and DAPI (blue) in Sham, Stroke, and Stroke + HDAC1 KD groups. Scale bar: 150 μm. Amplified images reveal a marked reduction in CD206 expression following stroke; however, no obvious further decrease was observed in the Stroke + HDAC1 KD group compared to the Stroke group. Scale bars: 50 μm. (B) Quantification of CD206^+^/Iba-1^+^ microglia, expressed as a percentage of total Iba-1^+^ cells. CD206 expression is significantly decreased after stroke in the Stroke and Stroke + HDAC1 KD groups (**p < 0.001, one-way ANOVA with Tukey’s *post hoc* test). *N* = 8 per group. Data are presented as mean ± SEM.

**Figure 3.**
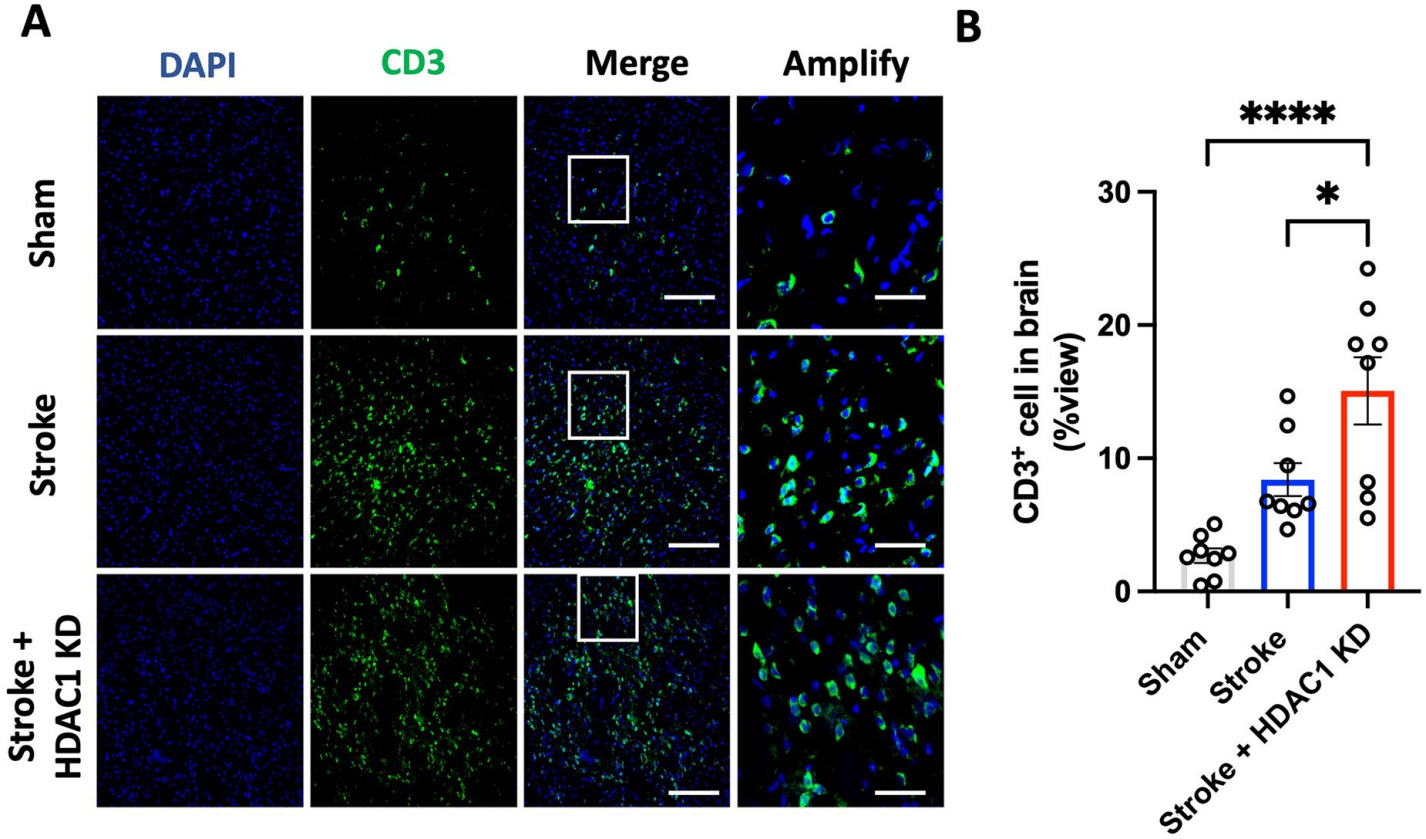
HDAC1 knockdown promotes T cell infiltration in the ischaemic brain. (A) Representative immunofluorescence images of brain sections stained for CD3 (green, T cell marker) and DAPI (blue, nuclei) in Sham, Stroke, and Stroke + HDAC1 KD groups. The Stroke group exhibits increased CD3^+^ cell presence compared to Sham controls, which is further enhanced in the Stroke + HDAC1 KD group. Scale bars: 150 μm; Scale bars: 50 μm in amplified views. (B) Quantification of CD3^+^ cell density, expressed as a percentage of total cells per field of view. CD3^+^ cell infiltration is significantly increased in the Stroke group and further elevated following HDAC1 knockdown (*p < 0.05, one-way ANOVA with Tukey’s *post hoc* test). *N* = 8 per group. Data are presented as mean ± SEM.

**Figure 4.**
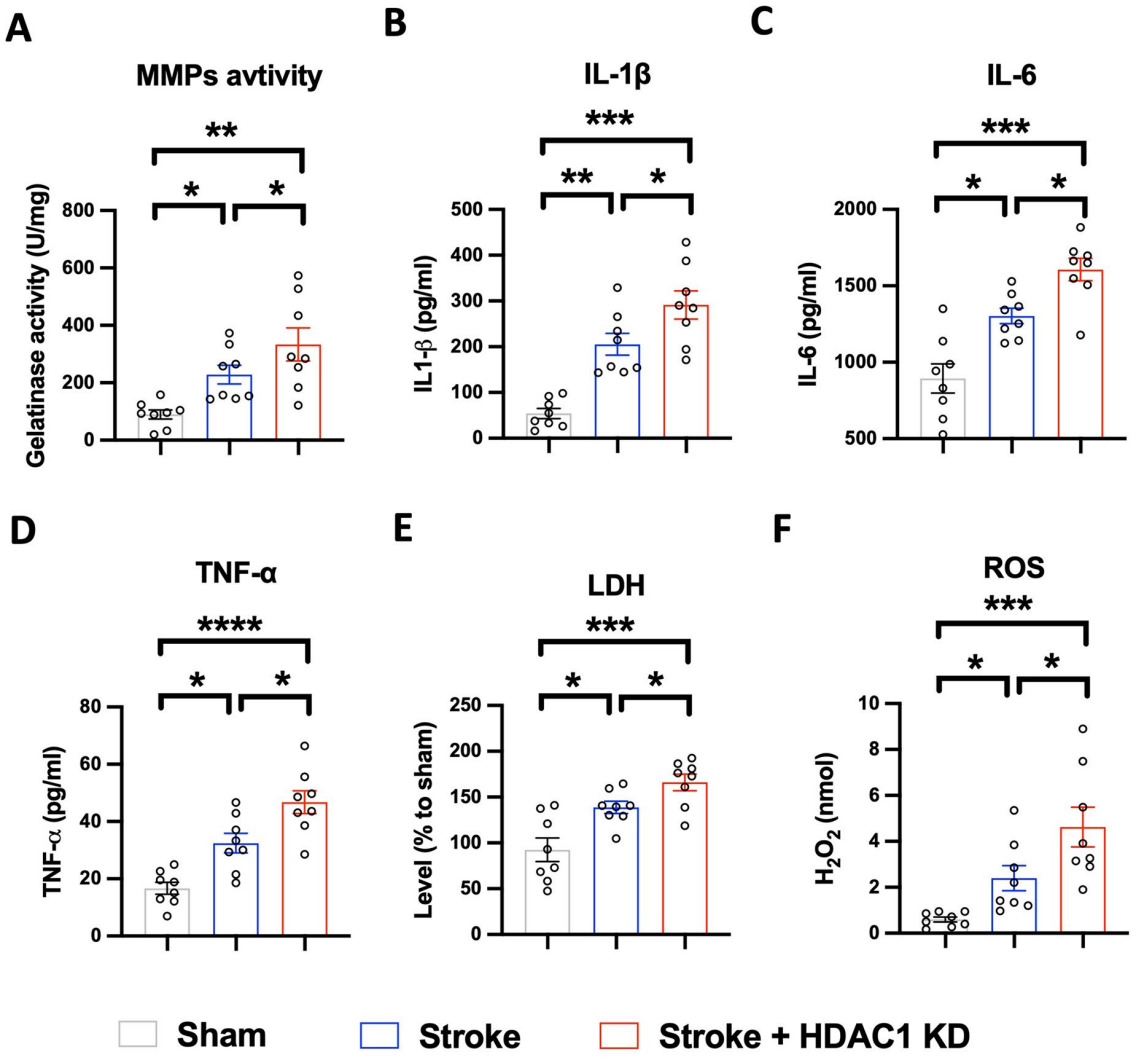
HDAC1 knockdown exacerbates stroke-induced neuroinflammation, oxidative stress, and neuronal injury. Bar graphs depict quantification of key inflammatory and injury markers in Sham, Stroke, and Stroke + HDAC1 KD groups. (A) MMPs activity measured by gelatinase assay. Stroke significantly increases MMPs activity, which is further enhanced by HDAC1 knockdown. (B and C) Pro-inflammatory cytokines IL-1β and IL-6 levels, showing a marked increase in the Stroke group, with further elevation in the Stroke + HDAC1 KD group. (D) TNF-α levels follow a similar pattern, with the highest expression observed in the Stroke + HDAC1 KD group. (E) LDH levels, indicating neuronal injury, are significantly increased following stroke and further elevated by HDAC1 knockdown. (F) ROS levels measured by H_2_O_2_ production demonstrate significantly elevated oxidative stress in the Stroke + HDAC1 KD group. *N* = 8 per group. Statistical significance: * *p* < 0.05, ** *p* < 0.01, ***p < 0.001, **** *p* < 0.0001 (one-way ANOVA with Tukey’s *post hoc* test). Data are presented as mean ± SEM.

### Immunofluorescent staining

Immunofluorescence staining was conducted following a previously established protocol. After deep anaesthesia, rats underwent transcardial perfusion with PBS followed by 4% paraformaldehyde (PFA). The brains were then excised, post-fixed, dehydrated, and embedded in optical coherence tomography (OCT) compound. Brain tissue sections were collected from bregma +2 mm to −4 mm, sliced into 10 μm sections, and systematically mounted onto slides to ensure consistent sample collection. Immunostaining was performed using the primary antibodies Iba-1 (Genetex GTX635363, Xinbei, Taiwan; 1:300), CD86 (Genetex GTX635363; 1:200), CD206 (Santa Cruz sc-58986; Dallas, Texas; 1:150), CD3 (GTX16669; 1:300). For visualization, AlexaFluor-conjugated secondary antibodies **(**Thermo Fisher Scientific, Waltham, MA, USA; 1:1000) were applied, and DAPI was used to counterstain cell nuclei. The sections were mounted with Dako Fluorescence Mounting Medium (Dako, Glostrup, Denmark). For quantification of immunoreactivity signals, a high-throughput automated imaging platform (ImageXpress^®^, Molecular Devices; San Jose, CA, USA) was used to capture images from the penumbra region of injured brains. Briefly, tissue sections were imaged using the ImageXpress Nano High-Throughput Screening (HTS) system. Image acquisition was controlled with MetaXpress software (version 6.7). Slides were mounted onto the imaging chamber, and images were acquired using a 20× objective following system calibration. Optimal focal plane and exposure time were automatically adjusted by the software prior to acquisition. Images were collected under standardized settings across all experimental groups. Image analysis was performed using MetaXpress software (version 6.7) with the Cell Scoring module. Nuclear identification was performed using DAPI (channel 1, baseline channel), and target signals were acquired using FITC or Texas Red (channel 2, depending on experimental design). Thresholds were applied for both channels to quantify double-positive cells.

### Western blot, ELISA and biochemical assays

Brain protein extracts were obtained from tissue samples within the bregma +3 mm to −1 mm region, which were sectioned using a brain slicer. The Western blot protocol followed our previous study [13]. The molecular weight ladder used in our experiment was the PageRuler^™^ Plus Prestained Protein Ladder (Thermo #26619). Primary antibodies used including: HDAC1 (Genetex GT1163; 1:1000), Phosph- and total NF-κB (Cellsignaling 3033S and 8242S; Danvers, MA; 1:1500), Phosph- and total Stat-1 (Cellsignaling 7649s; Genetext GTX13437; 1:500), Phosph- and total Stat-1 (Cellsignaling 7134s; Genetext GTX104616; 1:500), and GAPDH (Genetext, GTX100118; 1:5000). Reactive oxygen species (ROS) and lactate dehydrogenase (LDH) assays were conducted using commercial kits (BioVision K936-100-250 and K726; Milpitas, CA, USA), following the manufacturer’s instructions. ELISA kits for quantification of IL-1β, IL-6 and TNF-α were purchased from R&D Systems (RLB00 R6000B, and RTA00; Minneapolis, MN, USA). Matrix metalloproteinase 9 (MMP9) activity was assessed using an assay kit from Abcam (AB234057; Cambridge, UK). All experiments were conducted with fresh brain lysates, and procedures adhered strictly to the manufacturers’ protocols.

### Quantitative real-time PCR (qPCR)

Total RNA was extracted using the RNeasy Mini Kit (Qiagen, Cat. 74106, Hilden, Germany) according to the manufacturer’s instructions. RNA concentration and purity were assessed using a NanoDrop 2000 spectrophotometer (Thermo Fisher Scientific, Waltham, MA, USA). cDNA was synthesized using the PrimeScript RT Reagent Kit with gDNA Eraser (Takara, Cat. RR047A, Shiga, Japan). Quantitative real-time PCR was performed using TB Green^®^ Premix Ex Taq^™^ II (Takara, Cat. RR820A, Shiga, Japan) on a StepOnePlus^™^ Real-Time PCR System (Applied Biosystems, Thermo Fisher Scientific, USA). Relative gene expression was calculated using the 2^–ΔΔCt method, with Gapdh as the internal control. The primer sequences used were as follows: NFKBIA forward 5′-TACACCTTGCCTGTGAGCAG-3′ and reverse 5′-GACATCAGCACCCAAGGACA-3′; NFKB2 forward 5′-GGGGACTTCTCTCCCACAGA-3′ and reverse 5′-TTCCTCTGCACTTCCTCCTTG-3′; AP-1 forward 5′-GTGCCGAAAAAGGAAGCTGG-3′ and reverse 5′-GCTGCGTTAGCATGAGTTGG-3′; SAT1 forward 5′-CAGTGACATACTGCGGCTGA-3′ and reverse 5′-TGCAACCAGGCAGTGGTAAA-3′; MAP3K8 forward 5′-TGGAGTACATGAGCACTGGA-3′ and reverse 5′-TGCTGGCTCTTCACTTGCAT-3′; GAPDH forward 5′-AGGTGAAGGTCGGAGTCAAC-3′ and reverse 5′-TTCTCAGCCTTGACGGTGC-3′.

### Compound 5104434 treatment and functional evaluation

Compound 5104434 (ChemBridge, San Diego, CA, USA) was dissolved in DMSO (3 mg/mL stock) and administered by daily intraperitoneal injection (6 mg/kg/day) [14]. Sham and stroke groups received PBS with 1% DMSO as vehicle. Neurological function was assessed on post-stroke days (PSDs) 1, 3, and 7 using the modified neurological severity score (mNSS), adapted from the NSS [10]. Parameters included gait, body symmetry, climbing, turning, forelimb extension, circling, and sensory response; scores were summed to yield overall performance. Forelimb deficits were evaluated by the cylinder test [10]. Rats were placed in a transparent cylinder, and independent wall contacts with each forelimb were recorded. The impaired forelimb usage ratio was calculated as R/(L + R) × 100%. All rats underwent two days of pre-training to acclimate to the environment; non-adapted animals were excluded to reduce variability.

### Statistical analysis

All datasets were tested for normality and met the assumptions of normal distribution. Data are presented as mean ± SEM. Statistical comparisons were conducted using one-way ANOVA followed by Tukey’s post hoc test for multiple-group comparisons. Quantitative data were exported and further analysed using GraphPad Prism (version 9.0; GraphPad Software, San Diego, CA, USA) for statistical evaluation. A *p*-value < 0.05 was considered statistically significant.

## Results

### HDAC1 knockdown promotes pro-inflammatory microglial activation after stroke

**Figure 1.**
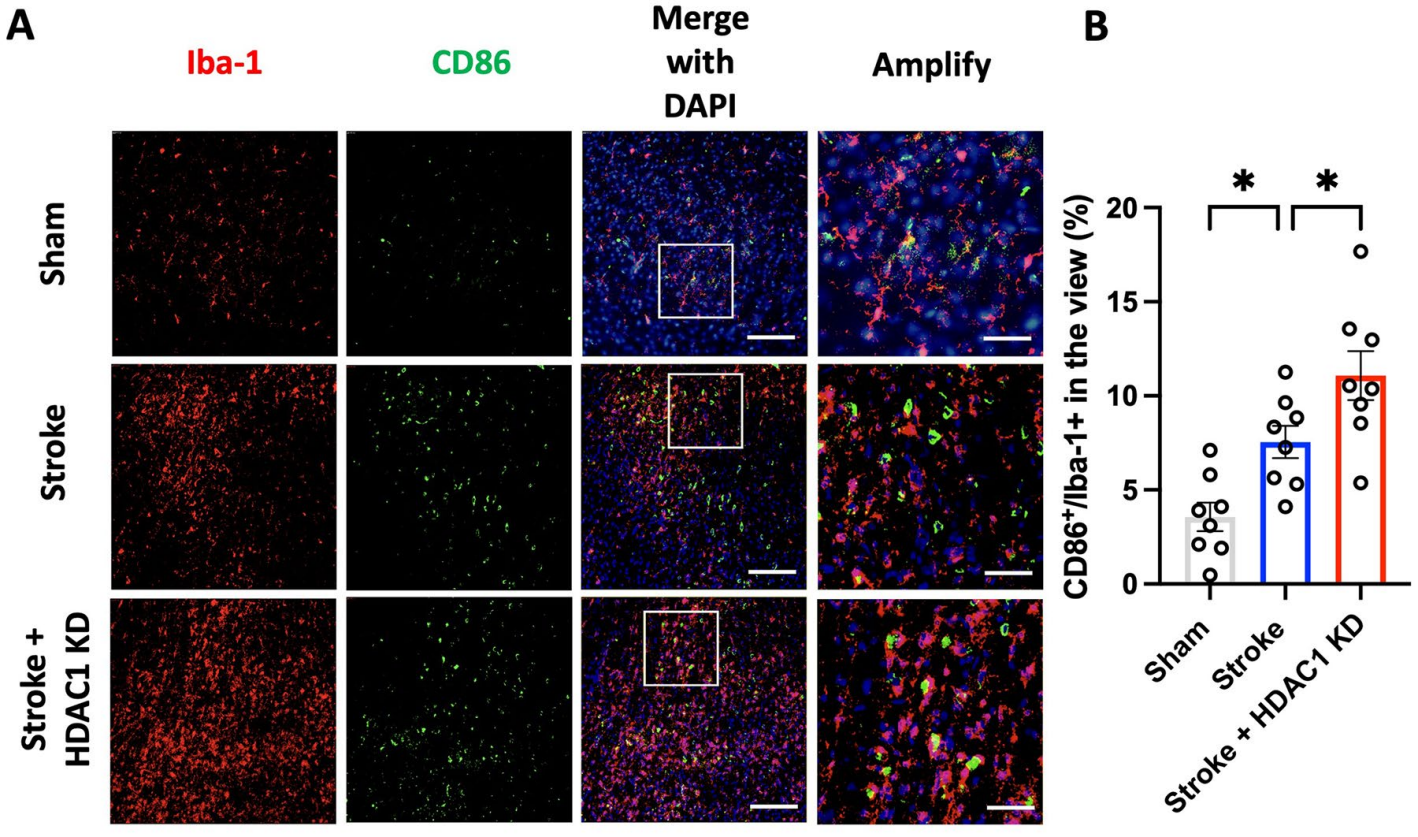
HDAC1 knockdown promotes pro-inflammatory microglial activation after stroke. (A) Representative immunofluorescence images of brain sections stained for Iba-1 (red, microglia), CD86 (green, M1 marker), and DAPI (blue, nuclei) in Sham, Stroke, and Stroke + HDAC1 KD groups. Scale bar: 150 μm. Amplified images highlight increased colocalization of CD86 with Iba-1 following stroke, which is further enhanced by HDAC1 knockdown. Scale bar: 50 μm. (B) Quantification of CD86^+^/Iba-1^+^ microglia, expressed as a percentage of total Iba-1^+^ cells. CD86 expression is significantly upregulated in the Stroke group and further increased following HDAC1 knockdown (*p < 0.05, one-way ANOVA with Tukey’s *post hoc* test). *N* = 8 per group. Data are presented as mean ± SEM.

To assess the role of HDAC1 in microglial activation following ischemic stroke, we adopt a rat stroke model of endothelin-1 injection and performed HDAC1 knock down in the cerebral cortex by concurrently stereo-microinjection of endothelin-1 and HDAC1 siRNA. To investigate the role of HDAC1 in microglial polarization following ischemic stroke, we examined the expression of pro-inflammatory (Figure 1) and anti-inflammatory (Figure 2) microglial markers in brain sections from Sham, Stroke, and Stroke + HDAC1 KD groups 3 days after brain ischemia induction. Immunofluorescence analysis of Iba-1 and CD86 revealed a significant increase in the proportion of CD86^+^/Iba-1^+^ cells in the peri-infarct region of the Stroke group compared to the Sham group (Figure 1(A,B)). This effect was further exacerbated in the Stroke + HDAC1 KD group, where CD86^+^ microglia were significantly more abundant compared to the Stroke group (*p* < 0.05). These results suggest that HDAC1 knockdown amplifies the pro-inflammatory microglial response after stroke. Conversely, analysis of Iba-1 and CD206 demonstrated a significant decrease in CD206^+^/Iba-1^+^ cells in the Stroke group compared to Sham controls, indicating a shift away from an anti-inflammatory phenotype (*p* < 0.001) (Figure 2(A,B)). Notably, this reduction was also marked in the Stroke + HDAC1 KD group. Taken together, these findings suggest that HDAC1 dysfunction drives microglia toward to a pro-inflammatory activation state, thereby contributing to heightened neuroinflammation following ischemic stroke.

### HDAC1 knockdown enhances CD3^+^ T cell infiltration in the ischaemic brain

In addition to microglial activation, post-stroke neuroinflammation is characterized by peripheral immune cell infiltration, including T lymphocytes [15]. To assess the impact of HDAC1 knockdown on T-cell recruitment, we performed immunofluorescence staining for CD3, a marker of T lymphocytes, in Sham, Stroke and Stroke + HDAC1 KD groups. As shown in Figure 3(A), CD3^+^ T cells were rarely observed in Sham animals. However, stroke significantly increased the presence of CD3^+^ cells in the peri-infarct region, suggesting T cell infiltration. This effect was further exacerbated in the Stroke + HDAC1 KD group, where a markedly greater proportion of CD3^+^ cells was observed compared to the Stroke group. Quantification of CD3^+^ cells (Figure 3(B)) confirmed this trend, with a significant increase in CD3^+^ cell density in the Stroke + HDAC1 KD group relative to both the Sham and Stroke groups (*p* < 0.05). These findings indicate that HDAC1 dysfunction promotes an exaggerated T cell response in the ischemic brain, further amplifying post-stroke neuroinflammation.

### HDAC1 knockdown exacerbates stroke-induced neuroinflammation and oxidative stress

To further investigate the impact of HDAC1 dysregulation on neuroinflammation and oxidative stress following stroke, we analysed the levels of key inflammatory cytokines (IL-1β, IL-6, and TNF-α), matrix metalloproteinases (MMPs) activity, lactate dehydrogenase (LDH), and reactive oxygen species (ROS) production in the brain tissue of Sham, Stroke, and Stroke + HDAC1 KD groups 3 days after stroke. As shown in Figure 4, stroke significantly increased MMP activity (Figure 4(A)), as evidenced by elevated gelatinase activity compared to the Sham group (*p* < 0.05). This increase was further exacerbated in the Stroke + HDAC1 KD group (*p* < 0.01)) (Figure 4(A)), suggesting that HDAC1 knockdown promotes extracellular matrix degradation, which is associated with increased blood-brain barrier (BBB) permeability and neuronal damage. Similarly, pro-inflammatory cytokines IL-1β, IL-6 and TNF-α were significantly upregulated in the Stroke group relative to Sham controls (*p* < 0.001). Notably, HDAC1 knockdown further elevated these cytokine levels (Figure 4(B–D)), particularly IL-1β (*p* < 0.0001), indicating an amplified neuroinflammatory response.

To assess the extent of cellular injury, we measured LDH levels, which were significantly elevated following stroke and further increased in the Stroke + HDAC1 KD group (*p* < 0.01) (Figure 4(E)). Additionally, oxidative stress, measured by H_2_O_2_ production, followed a similar trend, with significantly increased ROS levels in the Stroke group and an even greater surge in the Stroke + HDAC1 KD group (*p* < 0.001) (Figure 4(F)). These findings collectively suggest that HDAC1 dysfunction exacerbates stroke-induced neuroinflammation, enhances oxidative stress, and contributes to greater neuronal injury, further supporting its role in worsening post-stroke pathology.

### HDAC1 knockdown in vitro enhances pro-inflammatory while suppressing anti-inflammatory microglial activation

**Figure 5.**
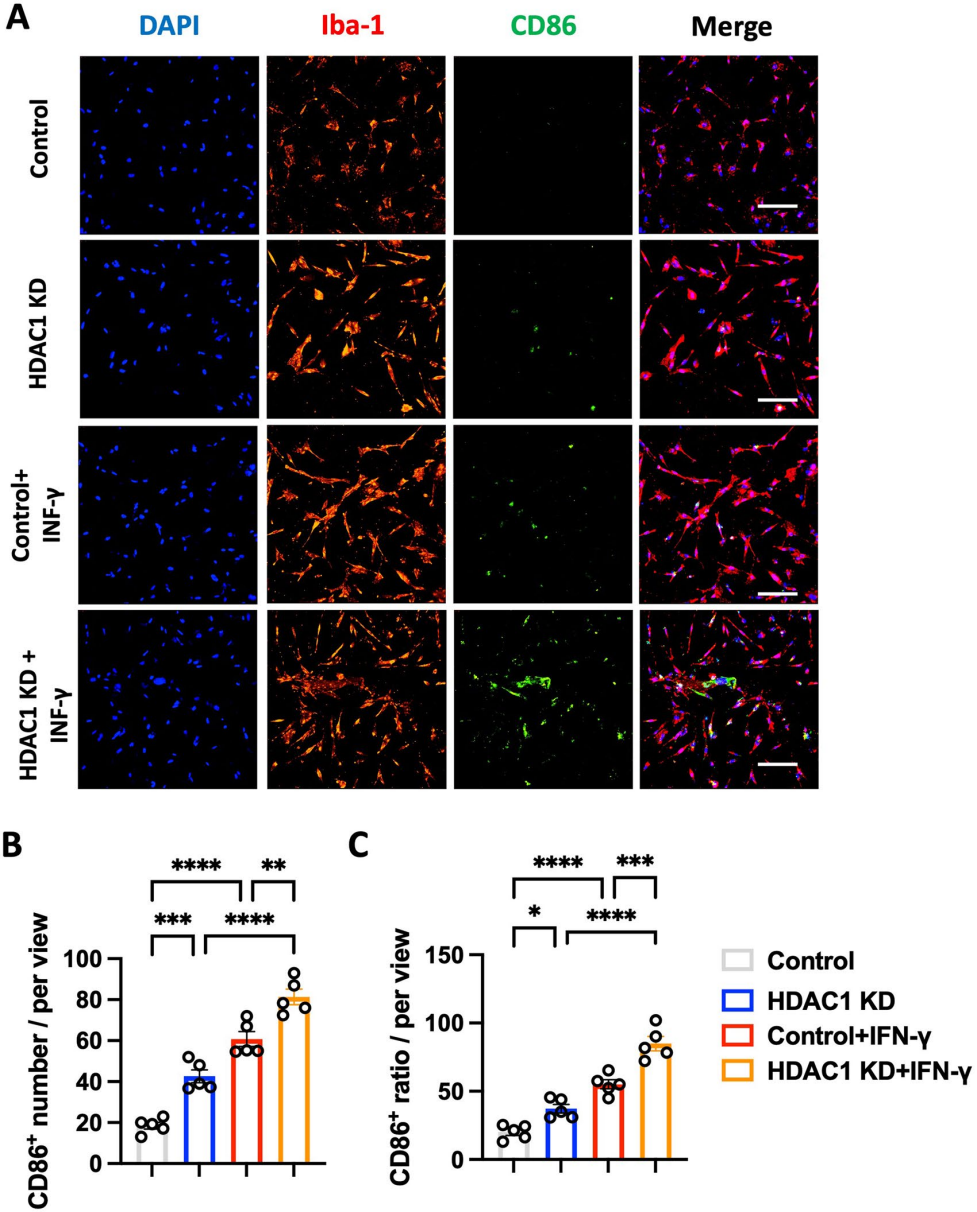
HDAC1 knockdown enhances pro-inflammatory microglial activation *in vitro*. (A) Representative immunofluorescence images of cultured human macrophage cell line-HMC3 stained for Iba-1 (red, microglial marker), CD86 (green, M1 marker), and DAPI (blue, nuclei) under different conditions: Control, HDAC1 KD, Control + IFN-γ, and HDAC1 KD + IFN-γ. Scale bars: 50 μm. (B) Quantification of M1 (CD86^+^) microglial cell number per field of view, showing a significant increase in CD86 expression with HDAC1 KD, IFN-γ treatment, and HDAC1 + IFN-γ treatment. (C) Percentage of CD86^+^ microglia per total microglial population, demonstrating that HDAC1 KD enhances M1 polarization, particularly in response to IFN-γ stimulation. *N* = 5 independent experiments. Statistical significance: * *p* < 0.05, ** *p* < 0.01, *** *p* < 0.001, **** *p* < 0.0001 (one-way ANOVA with Tukey’s *post hoc* test). Data are presented as mean ± SEM.

To further explore the role of HDAC1 in microglial functional shift, Interferon-γ (IFN-γ) was selected as a pro-inflammatory stimulus due to its well-characterized role in promoting pro-inflammatory activation of microglia/macrophages [16,17]. In the context of ischemic stroke, elevated levels of IFN-γ contribute to neuroinflammation by activating STAT1 and NF-κB signalling cascades [18,19]. Accordingly, we investigated the expression of pro-inflammatory and anti-inflammatory markers in a human macrophage cell line-HMC3 under control conditions, HDAC1 knockdown (HDAC1 KD), IFN-γ stimulation, and combined HDAC1 KD + IFN-γ treatment. As shown in Figure 5(A), control microglia displayed minimal CD86 expression. HDAC1 KD alone resulted in a moderate increase in CD86^+^ microglia, while IFN-γ treatment significantly induced CD86 expression in the control group. Notably, the HDAC1 KD + IFN-γ group exhibited the highest proportion of CD86^+^ microglia, suggesting that HDAC1 loss enhances microglial responsiveness to pro-inflammatory stimuli. Quantification of CD86^+^ microglia (Figure 5(B,C)) confirmed a significant increase in pro-inflammatory phenotype in the HDAC1 KD and control + IFN-γ groups compared to control (*p* < 0.05, *p* < 0.01, *p* < 0.001). The HDAC1 KD + IFN-γ group showed the greatest increase in pro-inflammatory activation state (*p* < 0.0001), supporting the notion that HDAC1 deficiency promotes a pro-inflammatory microglial phenotype.

**Figure 6.**
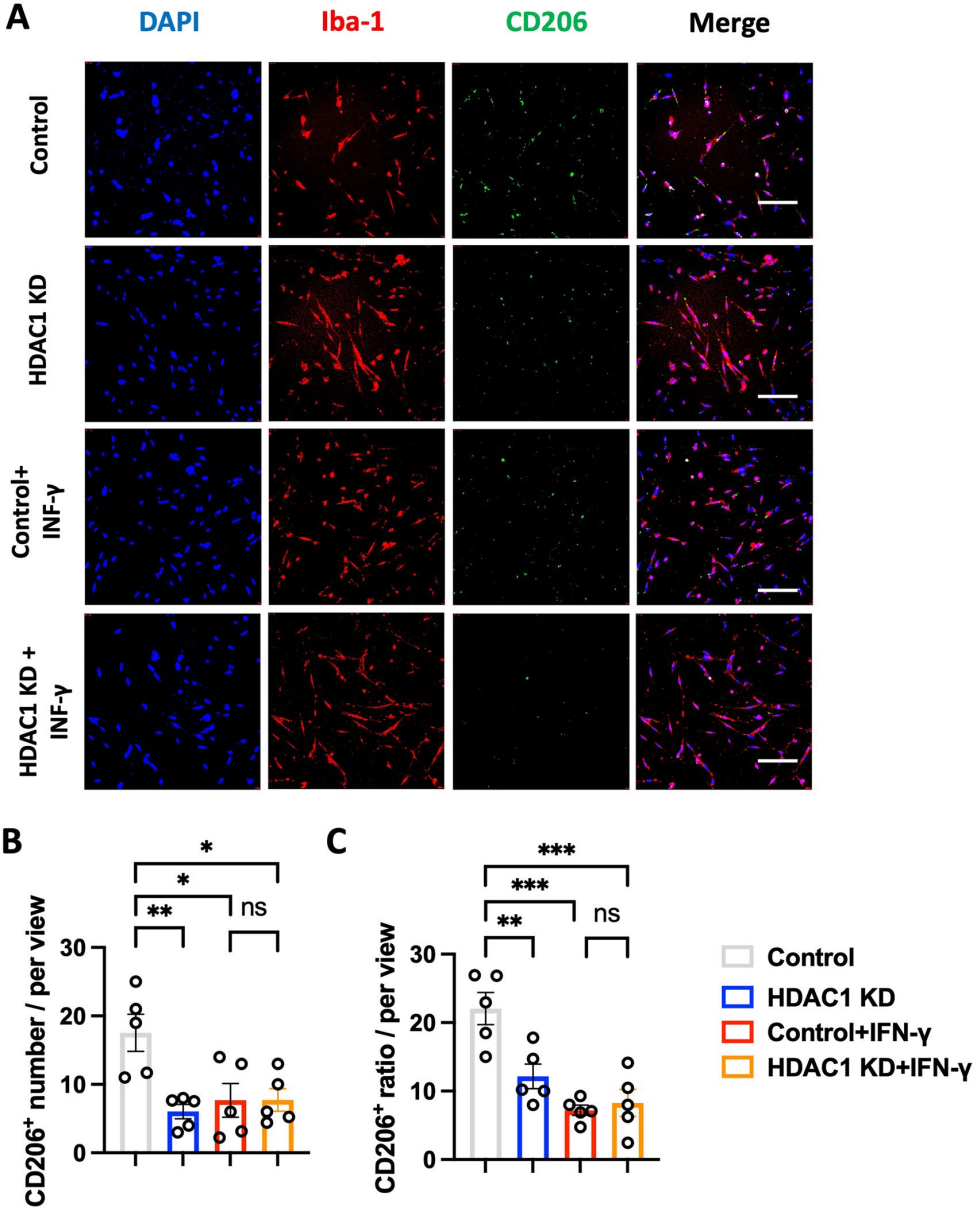
HDAC1 knockdown suppresses anti-inflammatory microglial activation *in vitro*. (A) Representative immunofluorescence images of cultured microglia stained for Iba-1 (red, microglial marker), CD206 (green, M2 marker), and DAPI (blue, nuclei) under different conditions: Control, HDAC1 KD, Control + IFN-γ, and HDAC1 KD + IFN-γ. Scale bars: 50 μm. (B) Quantification of M2 (CD206^+^) microglial cell number per field of view, showing a significant decrease in CD206 expression with HDAC1 KD and IFN-γ treatment. (C) Percentage of CD206^+^ microglia per total microglial population, indicating that HDAC1 KD suppresses M2 polarization, particularly in response to IFN-γ stimulation. *N* = 5 independent experiments. Statistical significance: * *p* < 0.05, ** *p* < 0.01, *** *p* < 0.001 (one-way ANOVA with Tukey’s *post hoc* test). Data are presented as mean ± SEM.

In addition, to examine whether HDAC1 knockdown affects anti-inflammatory microglial activation, we evaluated the expression of CD206, an anti-inflammatory microglial marker. As shown in Figure 6(A), CD206^+^ microglia were most abundant in the control condition. HDAC1 KD alone significantly reduced CD206 expression, and IFN-γ treatment also suppressed anti-inflammatory phenotype. Quantification of CD206^+^ microglia (Figure 6(B,C)) revealed a significant reduction in the HDAC1 KD and control + IFN-γ groups compared to control (*p* < 0.05, *p* < 0.01, *p* < 0.001). But, the HDAC1 KD + IFN-γ condition exhibited no further reduction of CD206^+^ microglia. These findings suggest that HDAC1 plays a critical role in microglial phenotypic state by promoting an anti-inflammatory phenotype and restraining excessive pro-inflammatory activation.

### HDAC1 knockdown activates pro-inflammatory transcriptional pathways

**Figure 7.**
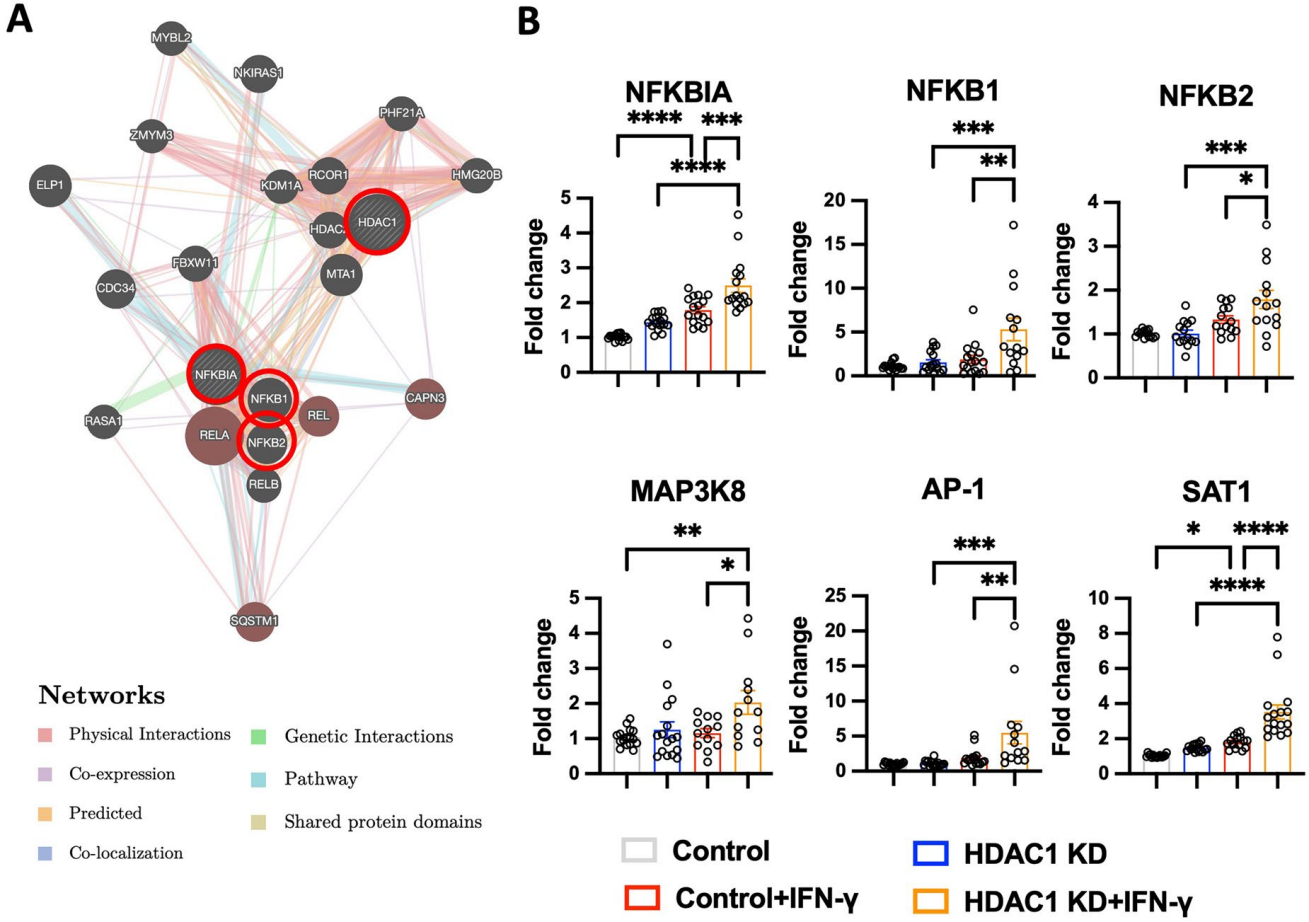
HDAC1 knockdown enhances pro-inflammatory transcriptional responses. (A) GeneMania network analysis showing predicted interactions of HDAC1 with transcriptional regulators involved in inflammatory signalling, including *NF-κBIA, NF-κB1* and *NF-κB2*. Physical interactions, genetic interactions, co-expression, and pathway connections are represented with different colours. (b) Real-Time PCR for gene expression analysis of *NF-κBIA, NF-κB1, NF-κB2, MAP3k8, AP-1,* and *SAT1* in microglia under Control, HDAC1 KD, Control + IFN-γ, and HDAC1 KD + IFN-γ conditions. Loss of HDAC1 significantly enhances the expression of NF-κB-related genes, as well as *AP-1* and *SAT1*, particularly in response to IFN-γ stimulation. *N* = 16 independent experiments. Statistical significance: * *p* < 0.05, ** *p* < 0.01, *** *p* < 0.001, **** *p* < 0.0001 (one-way ANOVA with Tukey’s *post hoc* test). Data are presented as mean ± SEM.

Our data reveal significant upregulation of TNF-α, IL-1β, and IL-6 (Figure 4), key cytokines associated with pro-inflammatory microglial phenotype. Our previous findings also showed that HDAC1 dysfunction promotes BBB disruption and neuroinflammation [13]. As a central regulator of pro-inflammatory gene expression, NF-κB activation drives pro-inflammatory activation by inducing cytokines and chemokines in microglia and other CNS cells [20,21]. Therefore, we further investigated NF-κB–associated signalling to elucidate its role in HDAC1-mediated neuroinflammatory responses following stroke. To elucidate the molecular mechanisms underlying HDAC1-mediated regulation of microglial activation, we performed a GeneMania network analysis to identify key interactors of HDAC1 (Figure 7(A)). The network highlights strong connections between HDAC1 and multiple transcription factors involved in inflammatory signalling, including *NF-κBIA, NF-κB1* and *NF-κB2*, suggesting a potential role of HDAC1 in modulating these pro-inflammatory pathways.

To experimentally validate these interactions, we assessed the gene expression levels of *NF-κBIA, NF-κB1, NF-κB2*, as well as NF-κB downstream key regulators: *MAP3K8, AP-1* and *SAT1*, in macrophage cell line-HMC3 under different conditions (Control, HDAC1 KD, Control + IFN-γ, and HDAC1 KD + IFN-γ). As shown in Figure 7(B), *NF-κBIA* expression was significantly upregulated following IFN-γ stimulation, and this effect was further amplified in HDAC1 KD + IFN-γ microglia (*p* < 0.001). Similarly, *NF-κB1* and *NF-κB2* expression was significantly increased in the HDAC1 KD + IFN-γ group compared to all other conditions (*p* < 0.05, *p* < 0.001), indicating enhanced activation of NF-κB signalling in the absence of HDAC1. Additionally, we observed a dramatic upregulation of *MAP3K8* and *AP-1* expression in the HDAC1 KD + IFN-γ group compared to controls (*p* < 0.01, *p* < 0.001), suggesting a heightened inflammatory response. Likewise, *SAT1*, a key regulator of IFN-γ-induced immune responses, exhibited significantly higher expression in the HDAC1 KD + IFN-γ condition compared to control (*p* < 0.0001), further supporting the role of HDAC1 in suppressing excessive inflammatory signalling. These findings suggest that HDAC1 plays a crucial role in restraining NF-κB, AP-1 and SAT1 activation, thereby regulating the extent of microglial inflammatory responses. Loss of HDAC1 function leads to enhanced transcriptional activation of these pro-inflammatory pathways, particularly under IFN-γ stimulation, reinforcing the concept that HDAC1 serves as a critical modulator of neuroinflammation.

### HDAC1 knockdown enhances NF-κB and STAT1/STAT3 signalling pathways

**Figure 8.**
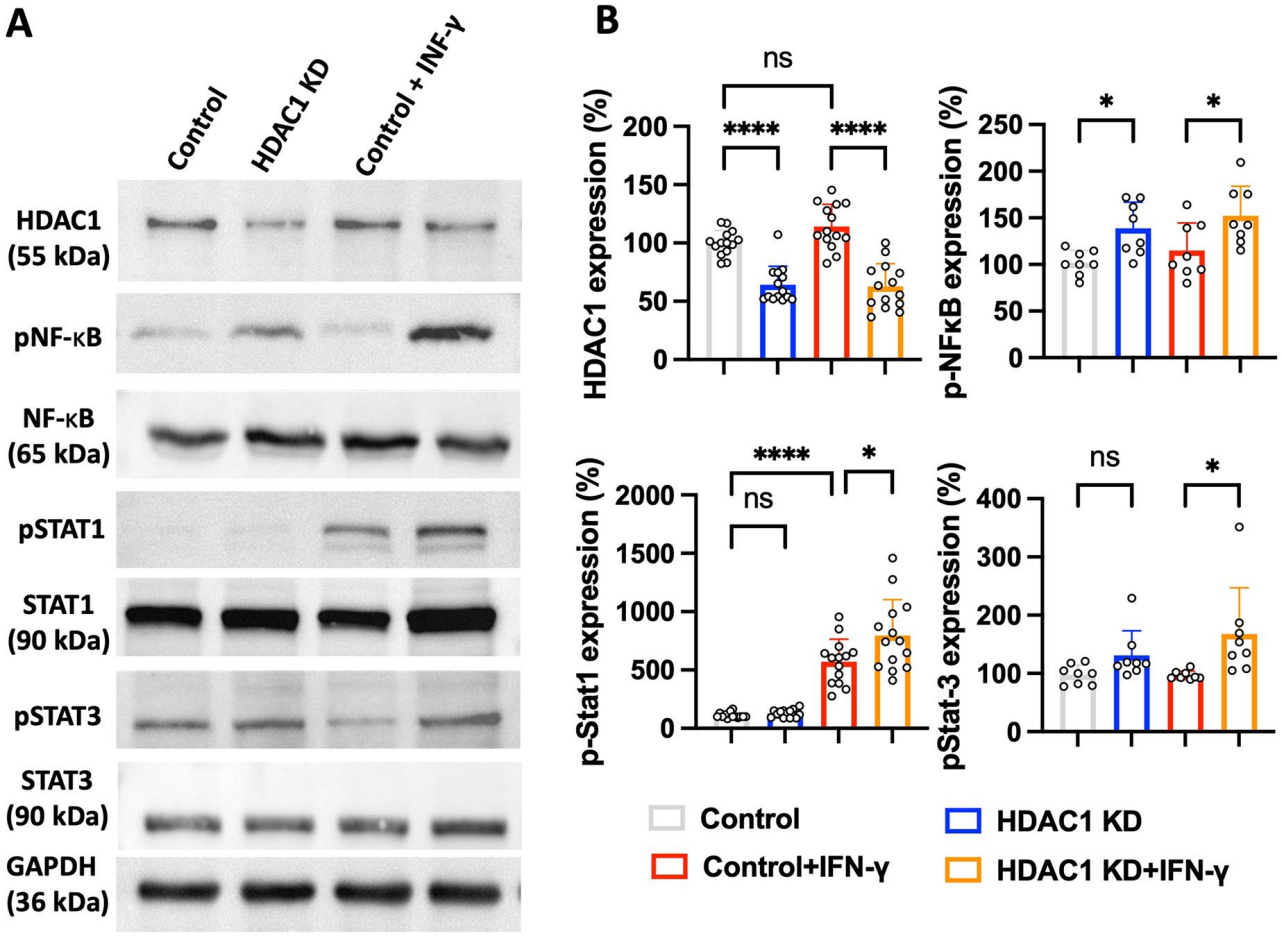
HDAC1 KD modulates NF-κB and STAT signalling pathways under IFN-γ stimulation. (A) Western blot analysis showing the expression levels of HDAC1, pNF-κB, total NF-κB, pStat1, total STAT1, pStat3 and total STAT3 across experimental groups: Control, HDAC1 KD, Control + IFN-γ, and HDAC1 KD + IFN-γ. GAPDH served as the loading control. HDAC1 KD resulted in altered expression and phosphorylation of key signalling proteins, particularly under IFN-γ stimulation. (B) Quantification of Western blot results, presented as normalized expression relative to control levels. HDAC1KD significantly reduced HDAC1 expression. Increased phosphorylation of NF-κB was observed in HDAC1 KD, with the highest levels in HDAC1 KD + IFN-γ. Phosphorylation of STAT1 was significantly upregulated under IFN-γ stimulation. HDAC1 KD increased STAT3 phosphorylation, and IFN-γ stimulation promoted its levels. *N* = 6 independent experiments. Data are presented as mean ± SEM, and statistical significance is indicated as follows: *p < 0.05, **p < 0.01, ***p < 0.001, ****p < 0.0001 (one-way ANOVA with Tukey’s *post hoc* test).

To further investigate the molecular mechanisms underlying HDAC1-mediated regulation of inflammatory responses, we assessed the protein expression levels of NF-κB, phosphorylated NF-κB (pNF-κB), STAT1, phosphorylated STAT1 (pSTAT1), STAT3, and phosphorylated STAT3 (pSTAT3) by Western blot analysis in microglia under different conditions (Control, HDAC1 KD, Control + IFN-γ, and HDAC1 KD + IFN-γ). As shown in Figure 8(A), HDAC1 protein expression was significantly reduced in the HDAC1 KD and HDAC1 KD + IFN-γ groups compared to the Control group (*p* < 0.0001), confirming the efficiency of HDAC1 knockdown. Loss of HDAC1 led to a significant increase in pNF-κB expression, indicating enhanced activation of NF-κB signalling (*p* < 0.05). This effect was further amplified by IFN-γ stimulation (*p* < 0.01), supporting the notion that HDAC1 functions as a negative regulator of NF-κB activation.

Similarly, pSTAT1 expression was significantly upregulated in both the Control + IFN-γ and HDAC1 KD + IFN-γ groups compared to Control (*p* < 0.0001). Notably, the HDAC1 KD + IFN-γ group did not exhibit elevation than Control + IFN-γ group.

In contrast, while pSTAT3 expression showed a modest increase in the HDAC1 KD, the highest expression was observed in the HDAC1 KD + IFN-γ condition (*p* < 0.05), further supporting the role of HDAC1 in regulating STAT3-dependent inflammatory signalling. Taken together, these findings suggest that HDAC1 negatively regulates NF-κB and STAT3 signalling pathways. Loss of HDAC1 leads to increased activation of these pro-inflammatory transcriptional regulators, particularly under inflammatory stimulation, exacerbating microglial inflammatory responses.

### Selective HDAC1 reactivation by compound 5104434 improves Neurological outcomes and attenuates microglial activation after stroke

**Figure 9.**
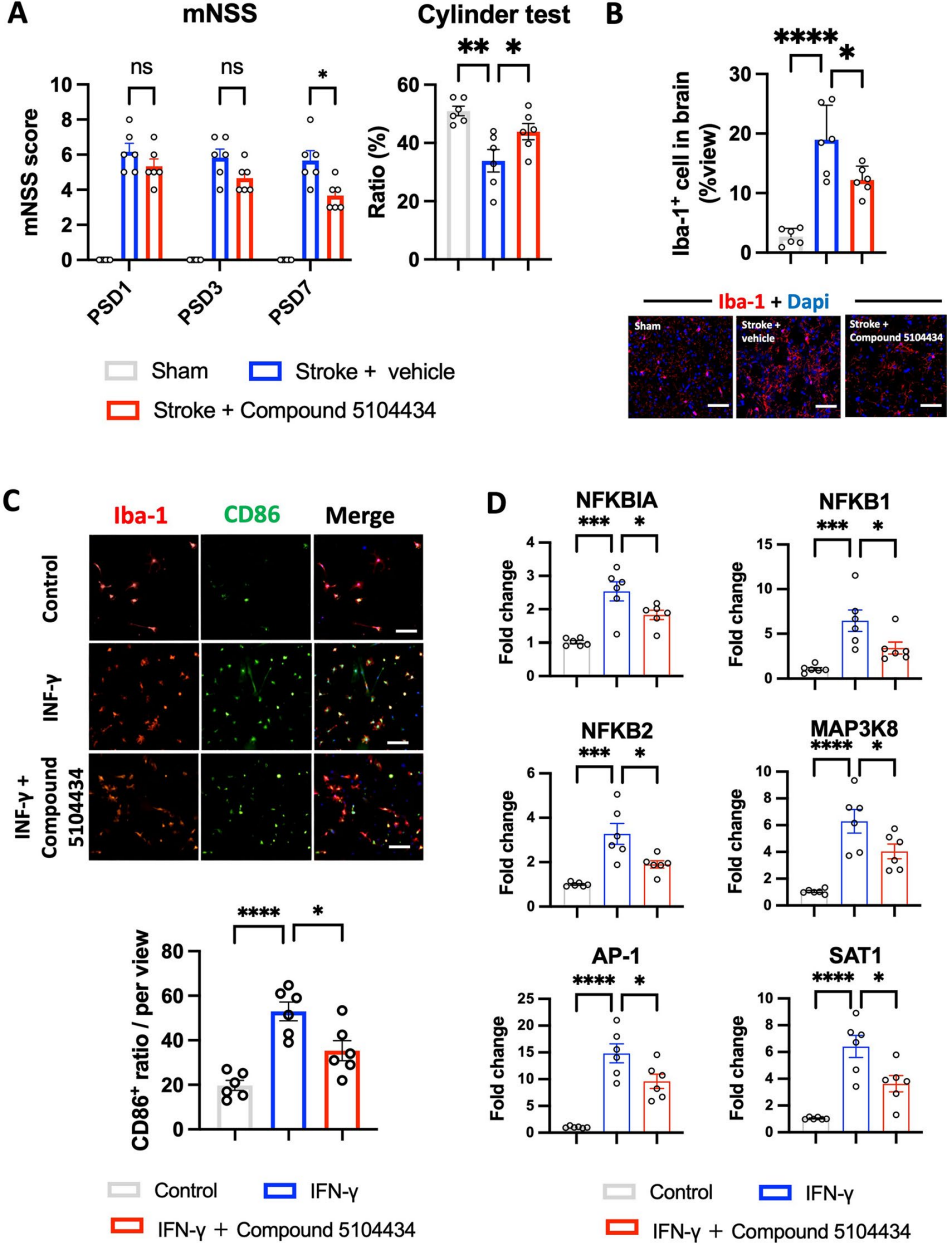
HDAC1 enzymatic reactivation by Compound 5104434 promotes functional recovery and suppresses NF-κB–driven microglial activation after stroke. (A) Neurological assessments showed mNSS scores (left) at PSD1, PSD3, and PSD7, with Compound 5104434 treatment significantly enhancing recovery compared to vehicle-treated stroke rats on PSD7. Cylinder test performance (right) was also improved on PSD7, indicating better forelimb function. *N* = 6 per group. (B) Microglial activation in peri-infarct cortex at PSD 7. Quantification of Iba-1^+^ cells (top) and representative immunofluorescence images (bottom; Iba-1, red; DAPI, blue). Scale bar: 100 μm. *N* = 6 per group. (C) Primary microglial polarization *in vitro*. Representative confocal images of Iba-1 (red) and CD86 (green) expression in control, IFN-γ, and IFN-γ + Compound 5104434 conditions (top). Quantification of CD86^+^ ratio per field (bottom). Scale bar:100 μm. *N* = 6 independent experiments per group. (D) Gene expression analysis by qPCR. Fold changes in *NFKBIA, NFKB1, NFKB2, MAP3K8, AP-1,* and *SAT1* following IFN-γ stimulation with or without Compound 5104434. *N* = 6 independent experiments per group. Data are mean ± SEM. *p < 0.05, **p < 0.01, ***p < 0.001, ****p < 0.0001 (One-way ANOVA with Tukey’s post hoc test).

Compound 5104434, a selective enzymatic reactivator of HDAC1 previously reported to exert beneficial effects in Alzheimer’s disease and experimental stroke models [7,10,14], was evaluated for its therapeutic efficacy in post-ischemic recovery. Stroke animals displayed persistent impairments in the modified Neurological Severity Score (mNSS) across 7 days compared with sham-operated rats. Treatment with Compound 5104434 significantly improved performance at PSD7, indicating enhanced recovery of neurologic function (Figure 9(A)). Similarly, cylinder test analysis revealed a significant reduction in forelimb asymmetry in Compound 5104434–treated rats compared with vehicle controls, indicating improved spontaneous forepaw use.

We next assessed microglial activation in peri-infarct regions at PSD 7. Quantification of Iba-1–positive cells demonstrated a marked increase after stroke, which was significantly attenuated by Compound 5104434 administration (Figure 9(B)). Confocal imaging confirmed reduced microglial density in treated brains, consistent with dampened neuroinflammatory responses. Furthermore, to further probe microglial proinflammatory status, we cultured primary microglial and examined CD86 expression under IFN-γ stimulation *in vitro*. IFN-γ robustly increased the ratio of Iba-1^+^/CD86^+^ pro-inflammatory microglia, while Compound 5104434 co-treatment significantly suppressed this response (Figure 9(C)). At the mechanistic level, quantitative PCR analyses revealed that IFN-γ stimulation upregulated canonical NF-κB–associated transcripts, including *NFKBIA, NFKB1, NFKB2, MAP3K8, AP-1* and *SAT1*. Compound 5104434 significantly downregulated these responses, consistent with reduced NF-κB pathway activation (Figure 9(D)). Collectively, these findings demonstrate that selective reactivation of HDAC1 by Compound 5104434 mitigates microglial-driven neuroinflammation and promotes functional recovery following ischemic stroke.

## Discussion

This study demonstrates that HDAC1 is a pivotal epigenetic regulator of neuroinflammation after ischemic stroke, acting at the intersection of microglial activation, peripheral immune infiltration, and transcriptional reprogramming. Using *in vivo* and *in vitro* approaches, we show that loss of HDAC1 function promotes a persistent pro-inflammatory state characterized by increased CD86^+^ microglia (Figures 1 and 5), elevated levels of pro-inflammatory cytokines (TNF-α, IL-1β, IL-6) (Figure 4), enhanced T-cell infiltration across a compromised blood–brain barrier (Figure 3), amplified oxidative stress, and greater neuronal injury (Figure 4), directly linking HDAC1 dysregulation to worsened functional outcomes. Importantly, our data establish causality, as HDAC1 knockdown exacerbated neuroinflammation, whereas selective HDAC1 reactivation with Compound 5104434 reversed these effects, improving behavioural recovery and suppressing NF-κB–driven transcriptional programs (Figure 9). This complementary loss- and gain-of-function evidence demonstrates that HDAC1 activity is required to suppress neuroinflammation and preserve neurological function. Mechanistically, HDAC1 deficiency enhanced NF-κB activation and upregulated downstream effectors including MAP3K8, AP-1 and SAT1, establishing a transcriptional environment that drives pro-inflammatory polarization, while STAT3 phosphorylation increased mainly under inflammatory stimulation, indicating that NF-κB is directly affected by HDAC1 loss and STAT3 responds more strongly in an inflammatory context (Figures 7 and 8). In summary, our findings suggest that HDAC1 regulates microglial activation and inflammatory signalling after stroke, with loss of HDAC1 enhancing pro-inflammatory responses and selective reactivation reducing neuroinflammation and improving recovery, supporting a role for HDAC1 in modulating post-stroke pathology.

Neuroinflammation after stroke is highly dynamic, and the timing of analysis is critical for interpreting the role of HDAC1 in this process. Our previous study demonstrated that HDAC1 inhibition at 24 h after stroke exacerbated gliosis and elevated IL-1β and TNF-α [13], underscoring its importance in the acute phase. In contrast, inhibition at two weeks post-stroke did not significantly affect BBB integrity or neuroinflammation, suggesting a time-dependent contribution of HDAC1. In the current study, we focused on post-stroke day 3 and 7 (PSD3, 7), as numerous reports indicate that neuroinflammation and M1-like microglial polarization reach a peak between PSD3 and PSD7, before shifting toward reparative phenotypes [22–24]. Thus, PSD3 represents a critical window to capture maximal inflammatory activity and assess the regulatory function of HDAC1. By targeting this pivotal early stage, our study provides mechanistic insight into how HDAC1 modulation influences microglial activation and the progression of neuroinflammation following ischemic stroke.

This microglial activation profile is not only molecularly defined but also functionally consequential—HDAC1 knockdown results in increased CD3^+^ T cell infiltration, elevated ROS and LDH levels, and enhanced MMPs activity, all of which converge on blood-brain barrier (BBB) dysfunction and secondary neuronal injury [25]. These results underscore that HDAC1 is integral to regulating not only intrinsic glial activation but also the recruitment and impact of peripheral immune cells within the ischemic brain.

This study offers important insight into how HDAC1 represses inflammatory transcriptional networks. A major finding is that HDAC1 suppresses NF-κB activation, one of the master regulators of immune and inflammatory gene expression. NF-κB is a pivotal transcription factor involved in stroke pathogenesis through regulation of inflammatory and apoptotic responses [26,27]. In ischemic stroke, reduced cerebral blood flow initiates cellular stress, leading to activation of several upstream signalling pathways, including Toll-like receptors (TLRs), cytokine receptors (e.g. TNF-α receptor), and interleukin-1 receptors (IL-1Rs). These pathways subsequently activate IκB kinase (IKK) complexes, resulting in phosphorylation and degradation of inhibitor κB (IκB). This degradation liberates NF-κB dimers (predominantly p65/p50), enabling their nuclear translocation [28]. Within the nucleus, NF-κB binds to specific DNA sequences and induces the transcription of genes involved in inflammation (e.g. IL-1β, TNF-α, IL-6, MCP-1), oxidative stress, endothelial activation, and apoptotic pathways, exacerbating neuronal damage and infarct size [29]. Thus, NF-κB signalling represents a critical target for therapeutic intervention aimed at mitigating inflammation and neurodegeneration following stroke. Here, our data showed that NF-κB signalling is significantly elevated in HDAC1-deficient microglia, even in the absence of exogenous inflammatory stimuli, suggesting that HDAC1 serves as a key brake on basal and inducible inflammatory tone.

Downstream of NF-κB, we observed the upregulation of key transcriptional regulators and signalling molecules that further propagate the inflammatory response, including *MAP3K8***,** *AP-1,* and *SAT1*. MAP3K8 is a pivotal upstream kinase linking NF-κB to MAPK/ERK signalling, and its induction enhances both ERK-mediated transcriptional activity and NF-κB amplification through IKK activation [30–32]. This creates a reinforcing loop in inflammation in which *MAP3K8* potentiates both NF-κB and *AP-1* signalling arms. Our data show that MAP3K8 is significantly upregulated in HDAC1-deficient microglia under inflammatory conditions, suggesting that HDAC1 loss may destabilize regulatory checkpoints upstream of both MAPK and NF-κB pathways.

AP-1, a dimeric transcription factor composed of Fos and Jun family proteins [33], is similarly upregulated in the HDAC1 KD + IFN-γ condition. AP-1 is well known to cooperate with NF-κB in driving the transcription of inflammatory genes such as IL-6, TNF-α, and MMPs [19]. Its activity is modulated by chromatin accessibility and co-factor recruitment—processes that HDAC1 has been implicated in. While the precise mechanisms remain to be clarified, HDAC1 may influence AP-1 activity indirectly through effects on MAPK signalling, or directly *via* modulation of chromatin structure or post-translational modification of AP-1 components.

SAT1 is another novel downstream target elevated in the context of HDAC1 deficiency. While SAT1 is traditionally viewed as a regulator of polyamine metabolism [34], recent studies implicate it in immune regulation, ferroptosis, and oxidative stress [35–37]. SAT1 activity promotes polyamine catabolism, generating reactive oxygen species [34], a byproduct that contributes to oxidative stress and NF-κB activation. The observed upregulation of SAT1 in HDAC1-deficient microglia suggests a potential link between HDAC1 loss and altered redox metabolism, which may play a role in amplifying neuroinflammatory responses.

In this study, we found that STAT1 phosphorylation was significantly induced by IFN-γ stimulation, whereas HDAC1 knockdown did not further increase pSTAT1 levels (Figure 8). STAT1 is a well-established mediator of IFN-γ–driven pro-inflammatory microglia phenotype, promoting the expression of pro-inflammatory genes such as *CXCL10*, *iNOS* and *IRF1* [38,39], which collectively amplify immune responses. In contrast, STAT3 activation was significantly enhanced in the HDAC1 KD + IFN-γ condition compared to IFN-γ treatment alone (Figure 8). While STAT3 is known to have both pro- and anti-inflammatory functions depending on the cellular context [40], it has also been associated with reactive gliosis, BBB permeability, and IL-6–mediated inflammation [41–43]. Notably, STAT3 activation in our study appeared more pronounced in HDAC1 KD + IFN-γ group than that HDAC1 KD only and Stroke + IFN-γ groups. These findings suggest that HDAC1 knockdown does not directly regulate STAT1 activation, but may instead influence STAT3-driven pathways, potentially through epigenetic mechanisms or context-dependent modulation of cytokine signaling.

We identified NF-κB as a central mediator of the inflammatory response following ischemia/reperfusion injury in this study. Consistent with previous findings, NF-κB activation drives the transcription of key pro-inflammatory cytokines, including IL-6 (Figure 4(C)). IL-6 signalling subsequently activates the JAK/STAT3 pathway, leading to sustained STAT3 phosphorylation and transcriptional activity [29,44]. While STAT3 is known to regulate glial reactivity and inflammatory gene expression [45,46], its precise role in microglial polarization remains context-dependent. Our findings suggest that HDAC1 deficiency may potentiate this inflammatory cascade by enhancing NF-κB activation and IL-6 production, thereby promoting sustained STAT3 signalling. Notably, this HDAC1–NF-κB–IL-6–STAT3 axis coincides with increased expression of pro-inflammatory markers, supporting the notion that HDAC1 loss contributes to pro-inflammatory microglial phenotype under ischemic conditions. Together, these results highlight a potential epigenetic-inflammatory circuit that amplifies neuroinflammation and may represent a therapeutic target for modulating microglial responses after stroke.

Previous studies have shown that HDAC1 expression increases after ischaemic stroke [47] and that pharmacological inhibition promotes a shift of microglia from M1 to M2, exerting neuroprotective effects [48]. We acknowledge these findings, but the discrepancies with our results likely reflect differences in specificity, upstream regulation, and experimental context. Broad HDAC inhibitors or herbal extracts affect multiple class I HDACs and signalling pathways, making it difficult to attribute effects specifically to HDAC1. Moreover, upstream regulators such as lncRNA H19 can modulate HDAC1 activity and yield divergent outcomes. Finally, variations in stroke models and timing of assessment may further explain inconsistent observations. In contrast, our selective siRNA knockdown reveals that HDAC1 intrinsically represses NF-κB–driven pro-inflammatory activation, highlighting the importance of cellular context when defining its role in ischemic stroke.

Collectively, these findings reframe HDAC1 as a function molecule in the post-stroke brain—one that preserves neuronal viability through genome maintenance and simultaneously restrains innate immune activation through epigenetic repression of NF-κB signalling. The identification of HDAC1 as a suppressor of pro-inflammatory microglia phenotype expands its therapeutic relevance from a neuronal survival factor to a broader modulator of neuroimmune crosstalk. This is particularly relevant in the context of ischemic stroke, where the inflammatory response plays a pivotal role in secondary brain injury. Moreover, given the involvement of microglial activation in other neurodegenerative conditions such as Alzheimer’s disease, Parkinson’s disease, and multiple sclerosis, the implications of this work likely extend beyond stroke to other CNS pathologies characterized by chronic neuroinflammation.

## Conclusion

This study identifies HDAC1 as a key epigenetic regulator that suppresses pro-inflammatory microglia activation and neuroinflammation following ischemic stroke. HDAC1 deficiency amplifies NF-κB signaling and its downstream effectors—including *MAP3K8, AP-1,* and *SAT1*—while enhancing pro-inflammatory cytokine expression, oxidative stress, immune cell infiltration, and p-STAT3 activation under inflammatory stimulation. Selective reactivation of HDAC1 enzymatic activity improves functional recovery and mitigates neuroinflammation. These findings redefine HDAC1 as a key modulator of neuroimmune interactions with therapeutic potential in stroke and neuroinflammation.

## Supplementary Material

Supplemental Material

Suppl fig 4 600dpi.png

ARRIEVE Author Checklist.pdf

Suppl fig 3 600dpi.png

Suppl fig 1 600dpi.png

Suppl fig 5 600dpi.png

Suppl fig 2 600dpi.png

## Data Availability

All data supporting the findings of this study are presented within the article (Figure 1–8). Additional data relevant to this work are available from the corresponding author (C.C. Wu) upon reasonable request.
