# Supplementary material for: HDAC1 dysregulation promotes pro-inflammatory microglial activation and aggravates post-stroke neuroinflammation
Source: Ann Med. 2025 Dec 12;57(1):2597624. doi: 10.1080/07853890.2025.2597624 (PMC12704127; doi:10.1080/07853890.2025.2597624)
Supplement: Supplemental Material [file IANN_A_2597624_SM5208.docx]

**Supplementaory Information**

**HDAC1 Dysregulation Promotes Pro-inflammatory Microglial Activation and Aggravates Post-stroke Neuroinflammation**

Jui-Shen Chen^1, 2, #^, Hao-Kuang Wang^1, 2, #^, Yu-Ting Su^3^, Yu-Cheng Ho^4^, Cheng-Loong Liang^1, 4^, Yung-Kuo Lee^5, 6, 7, 8^, Tian-Huei Chu^5, 6, 7, 8^, Yun-Shin Lin^9^, Cheng-Chun Wu^2, 4*^

^1^ Department of Neurosurgery, E-DA Hospital, I-Shou University, Kaohsiung City, Taiwan.

^2^ Graduate Institute of Medicine, College of Medicine, I-Shou University, Kaohsiung City, Taiwan.

^3^ Department of Obstetrics and Gynecology, Kaohsiung Chang Gung Memorial Hospital and Chang Gung University College of Medicine, Kaohsiung City, Taiwan.

^4^ School of Medicine, College of Medicine, I-Shou University, Kaohsiung City, Taiwan.

^5^ Medical Laboratory, Medical Education and Research Center, Kaohsiung Armed Forces General Hospital, Kaohsiung City, Taiwan.

^6^ Division of Experimental Surgery Center, Department of Surgery, Tri-Service General Hospital, National Defense Medical University, Taipei 11490, Taiwan

^7^ School of Medicine, National Defense Medical University, Taipei, 11490, Taiwan

^8^ Institute of Medical Science and Technology, National Sun Yat-sen University, Kaohsiung 80424, Taiwan

^9^ Department of Psychiatry, Kaohsiung Armed Forces General Hospital, Kaohsiung city, Taiwan.

^#^ Equal contribution

^*^ Corresponding author

**Supplementary Figure 1. Validation of HDAC1 knockdown in vivo.** Western blot analysis showing HDAC1 protein expression in rat cortex following in vivo knockdown at post surgery day 3 using different siRNA assays. HDAC1 protein (~55 kDa) was reduced by several siRNA sequences compared with negative control. Actin (~45 kDa) was used as a loading control. Rat HDAC1 siRNAs were obtained from Thermo in vivo (Thermo Fisher Scientific HDAC1 siRNAs: Catalog #4457308 s150966, s150967, s150968, s171844 and Catalog #4390771 s119557; Negative control: Catalog #4457287). The target sequences, target exons, and siRNA locations are available on the manufacturer’s official product webpage, referenced by the catalog numbers and assay IDs provided in this study.

**Supplementary Figure 2. Time-dependent HDAC1 knockdown in rat brain.** Western blot analysis of HDAC1 protein expression in brain lysates collected at post-stroke day (PSD) 3, 5, 7, and 14 after in vivo knockdown using HDAC1 siRNA (Thermo Fisher Scientific; Catalog #4457308, Assay s150967). A negative control siRNA (Catalog #4457287) was used for comparison. HDAC1 (~55 kDa) levels were reduced in HDAC1 KD samples relative to negative controls across time points. Actin (~45 kDa) was used as a loading control.

**Supplementary Figure 3. Specificity of HDAC1 knockdown in rat brain.** Western blot analysis of HDAC2 (~55 kDa) and HDAC3 (~49 kDa) protein expression following in vivo HDAC1 knockdown. Brain lysates from sham control and rats injected with negative control siRNA, or HDAC1 KD siRNA (Thermo Fisher Scientific; Catalog #4457308, Assay s150967) were analyzed. No significant changes were observed in HDAC2 or HDAC3 levels, confirming the specificity of HDAC1 knockdown. GAPDH (~36 kDa) was used as a loading control.

**Supplementary Figure 4. Validation of HDAC1 knockdown in microglia by immunofluorescence staining.** Representative images of brain sections from sham, stroke, and stroke + HDAC1 knockdown (KD) groups stained with DAPI (blue), Iba-1 (red, microglial marker), and HDAC1 (green). In sham animals, HDAC1 expression is readily detectable in Iba-1⁺ microglia. Stroke increased Iba-1⁺ microglial activation, accompanied by HDAC1 expression. In contrast, HDAC1 siRNA treatment (Thermo Fisher Scientific; Catalog #4457308, Assay s150967) markedly reduced HDAC1 immunoreactivity in Iba-1⁺ cells. Merged panels show colocalization of signals, with amplified insets (rightmost column) highlighting HDAC1 reduction in microglia following siRNA-mediated knockdown. Scale bar: 200 μm.

**Supplementary Figure 5. Validation of HDAC1 knockdown in human macrophage cell line HMC3.** Western blot analysis of HDAC1 protein expression (~55 kDa) in HMC3 cells transfected with HDAC1 siRNAs (Thermo Fisher Scientific, Catalog #4392420; Assay IDs s73 and s74; negative control: Catalog #430843). Compared with control and siRNA negative control groups, HDAC1 siRNA S74 effectively reduced HDAC1 protein levels. Actin (~45 kDa) was used as a loading control. The target sequences, target exons, and siRNA locations are available on the manufacturer’s official product webpage, referenced by the catalog numbers and assay IDs provided in this study.
