# Supplementary figures and images for: HDAC1 dysregulation promotes pro-inflammatory microglial activation and aggravates post-stroke neuroinflammation
Source: Ann Med. 2025 Dec 12;57(1):2597624. doi: 10.1080/07853890.2025.2597624 (PMC12704127; doi:10.1080/07853890.2025.2597624)

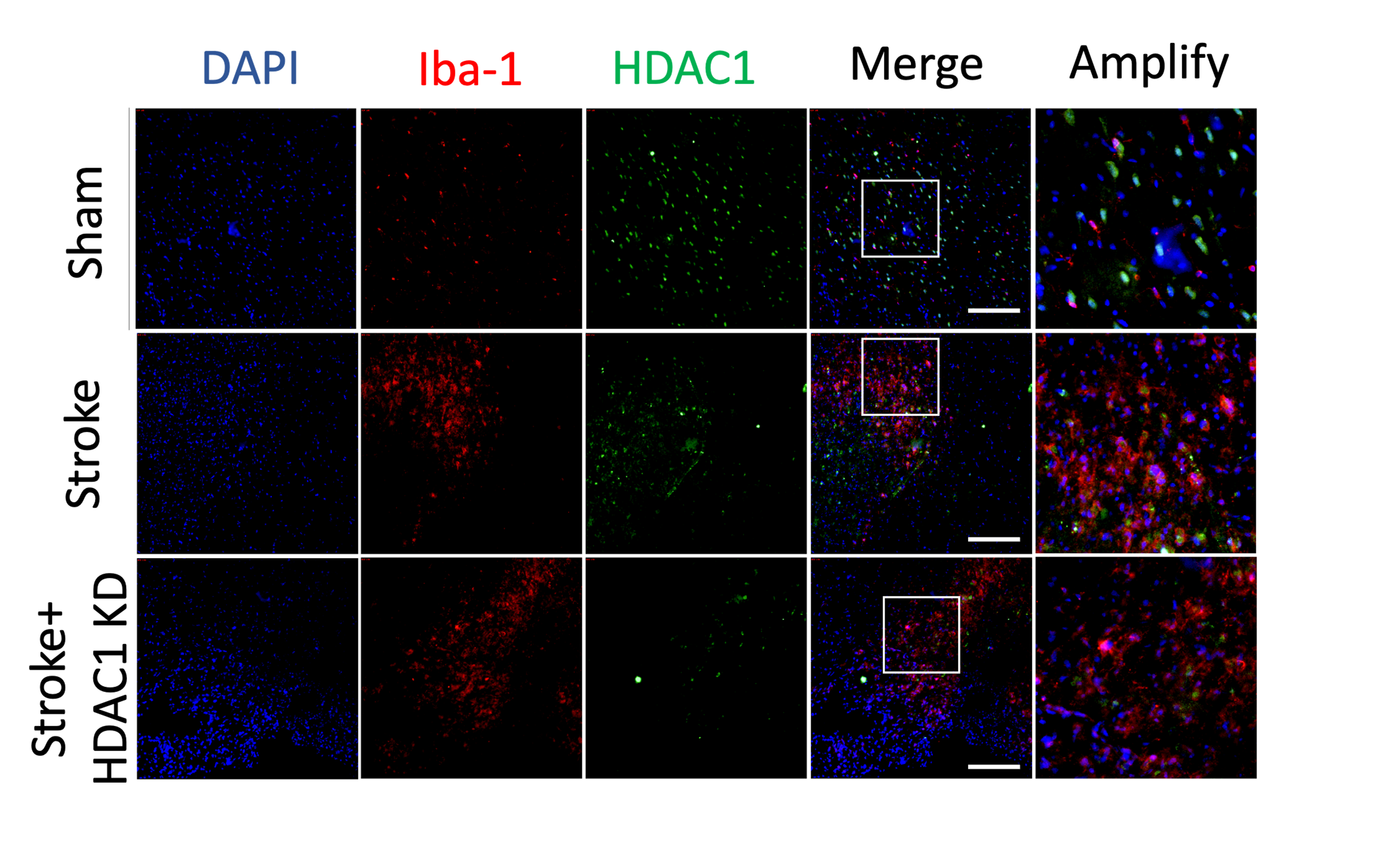

Supplement: Suppl fig 4 600dpi.png [file IANN_A_2597624_SM6557.png]

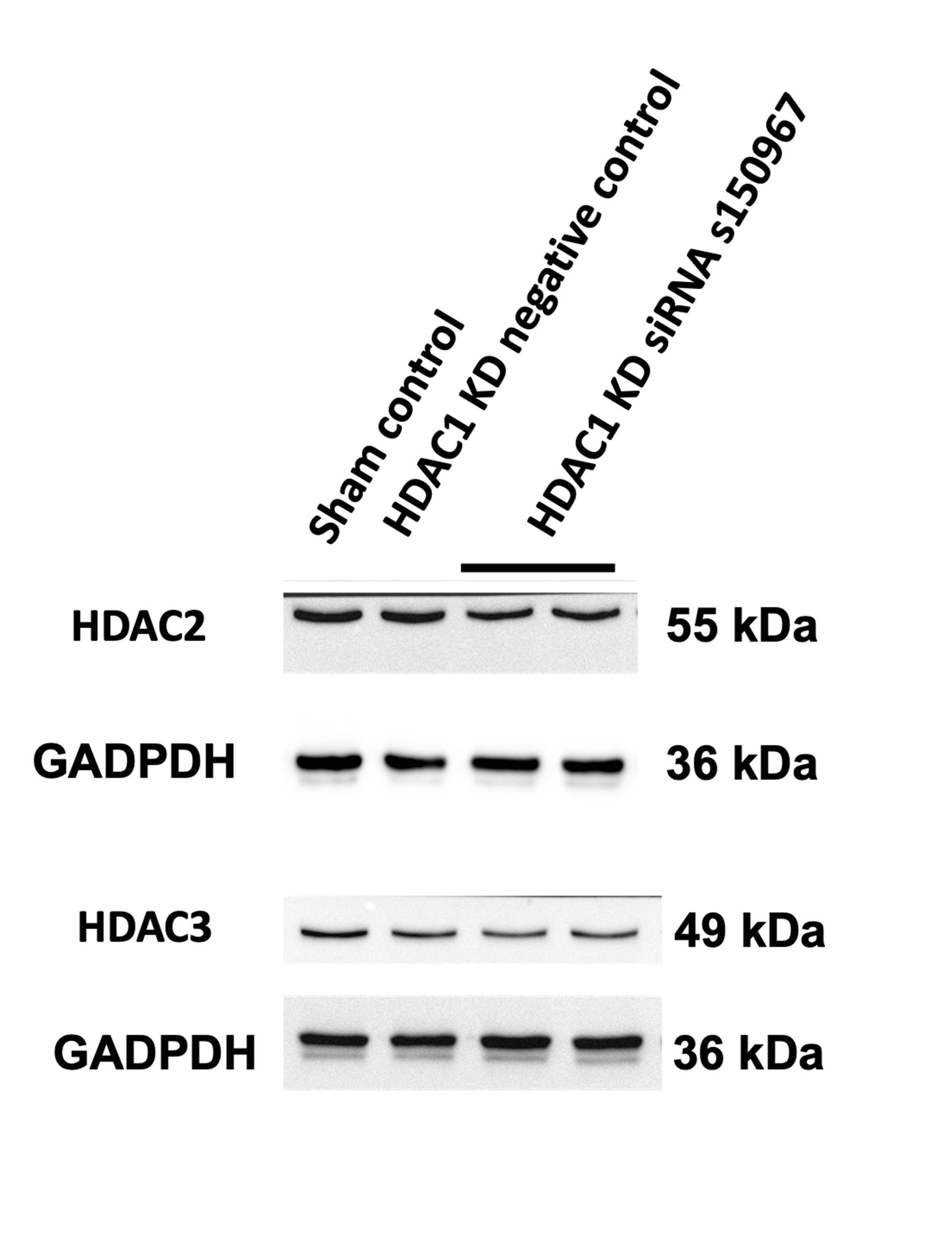

Supplement: Suppl fig 3 600dpi.png [file IANN_A_2597624_SM6555.png]

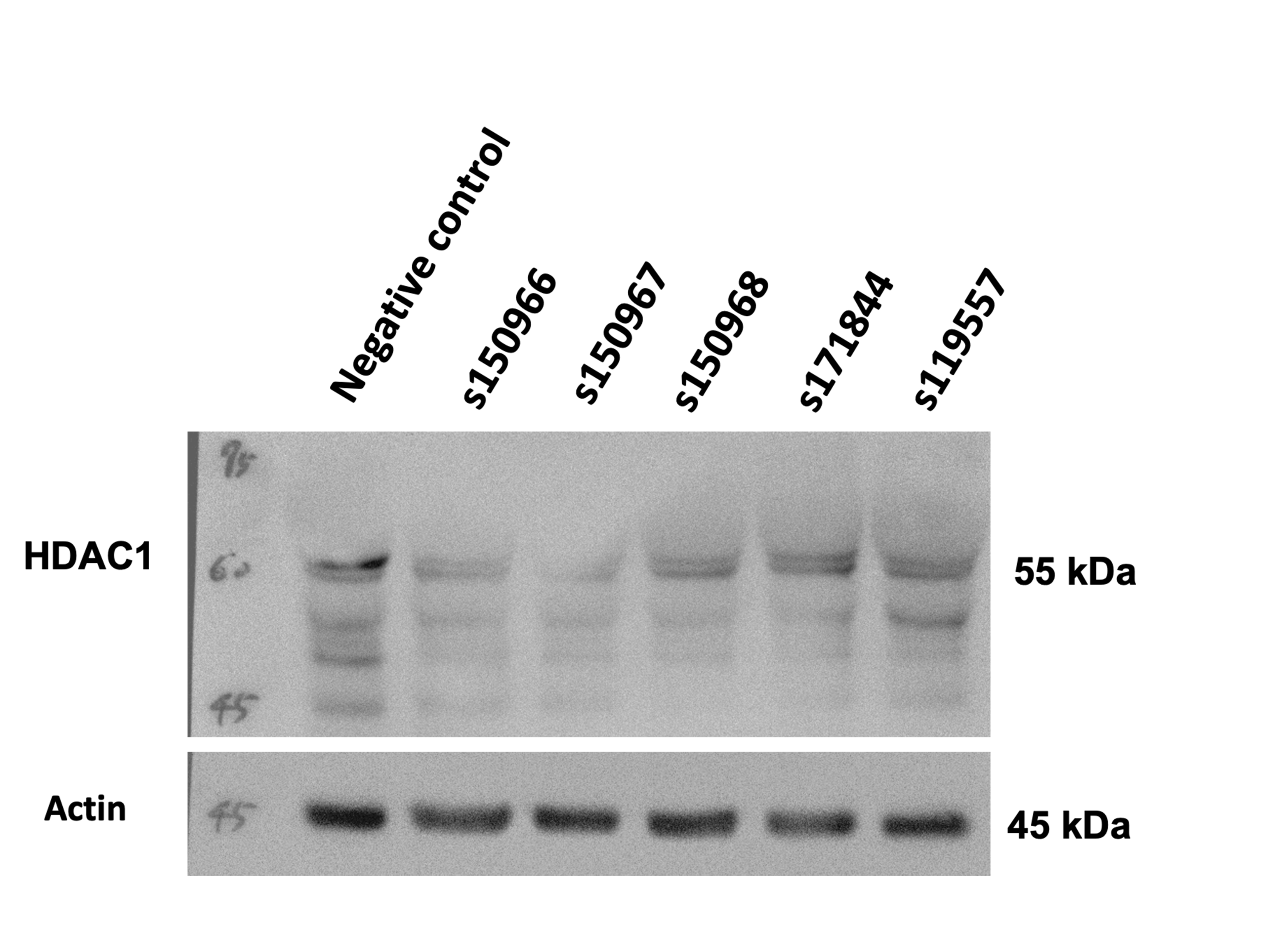

Supplement: Suppl fig 1 600dpi.png [file IANN_A_2597624_SM6554.png]

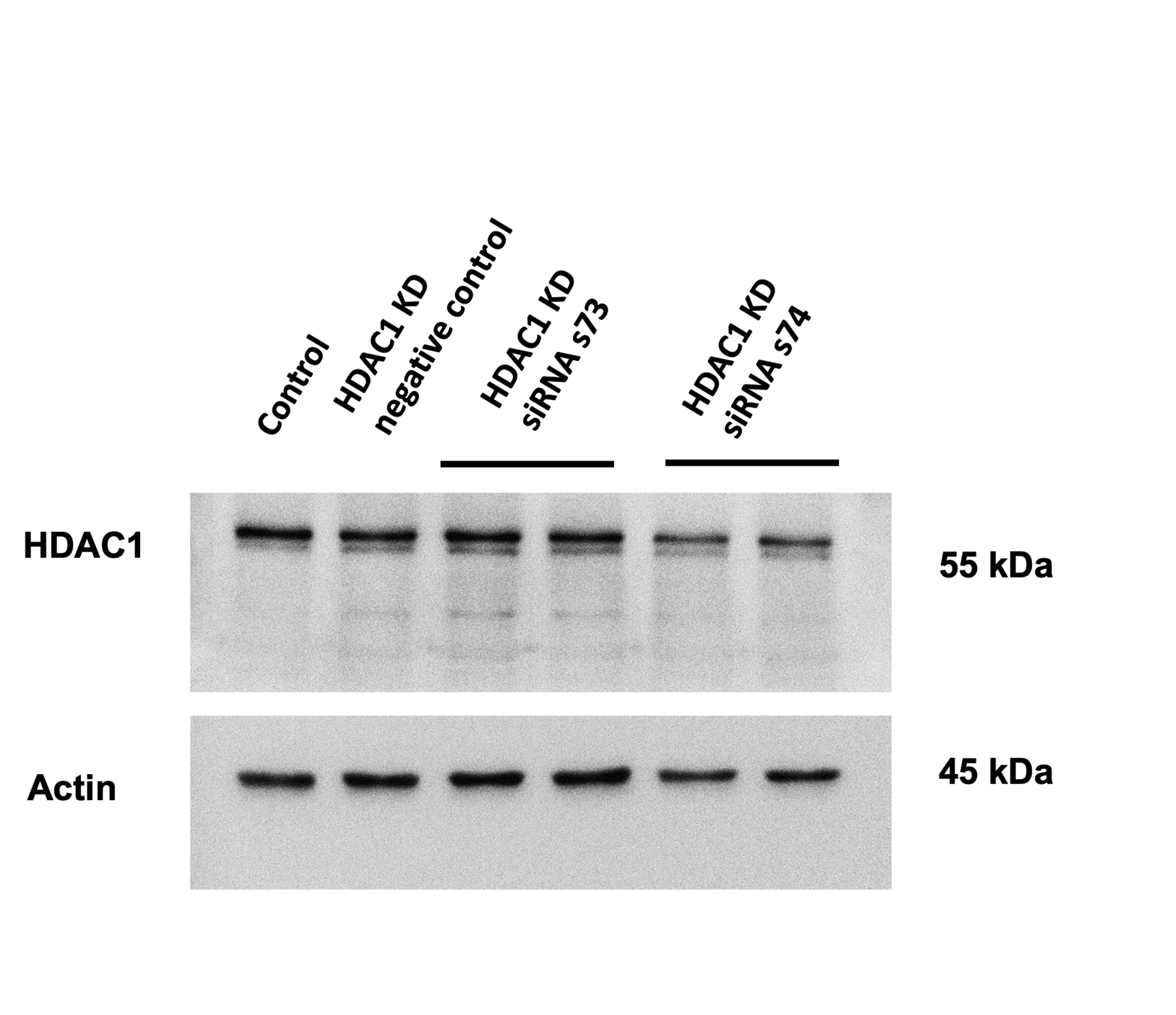

Supplement: Suppl fig 5 600dpi.png [file IANN_A_2597624_SM6553.png]

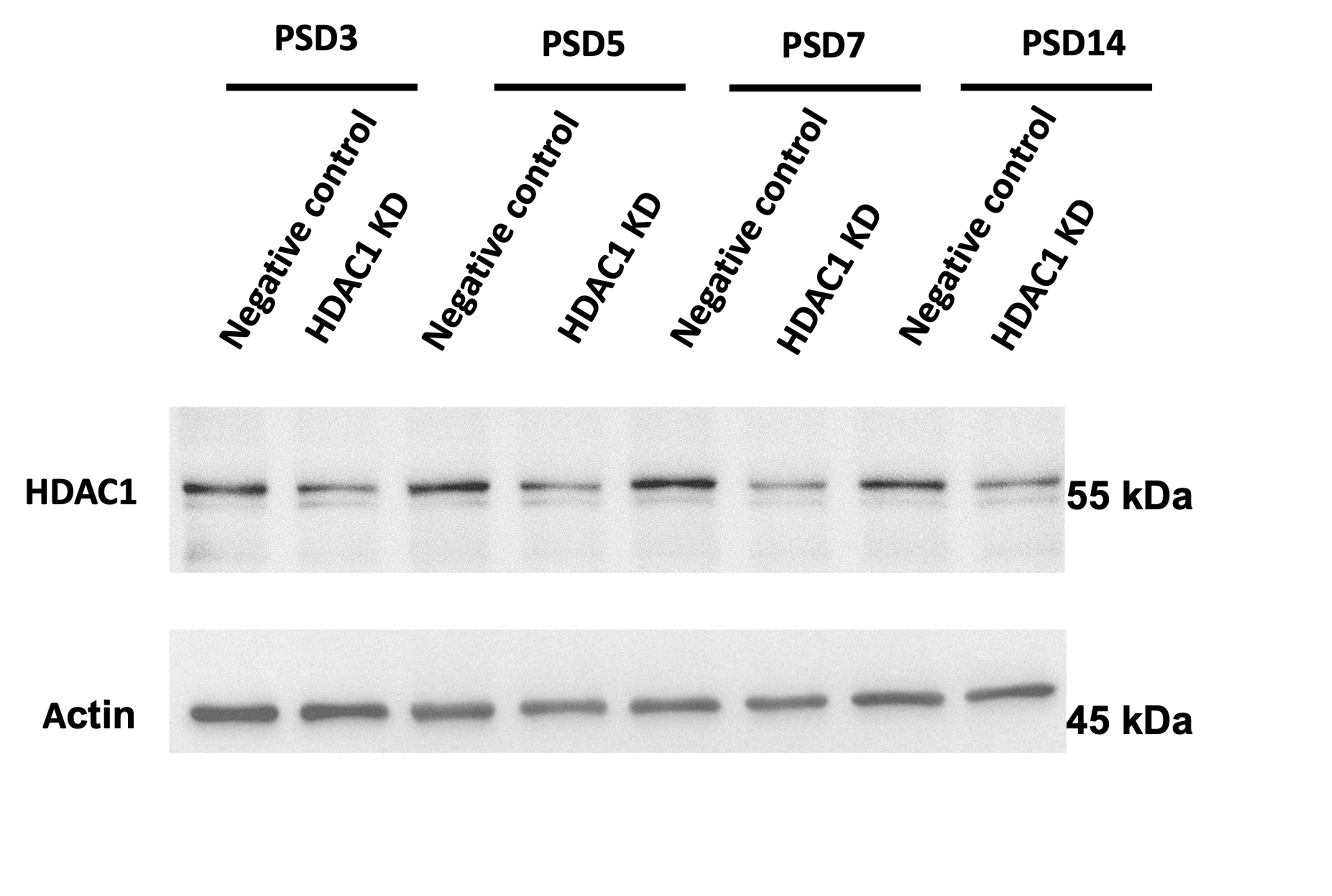

Supplement: Suppl fig 2 600dpi.png [file IANN_A_2597624_SM6552.png]
